# Chlorophyte aspartyl aminopeptidases: Ancient origins, expanded families, new locations, and secondary functions

**DOI:** 10.1371/journal.pone.0185492

**Published:** 2017-10-12

**Authors:** Sang-Youl Park, Melissa A. Scranton, Jason E. Stajich, Ashley Yee, Linda L. Walling

**Affiliations:** 1 Department of Botany and Plant Sciences, Center for Plant Cell Biology, University of California, Riverside, California, United States of America; 2 Department of Plant Pathology and Microbiology, Center for Plant Cell Biology, University of California, Riverside, California, United States of America; University of Maryland, UNITED STATES

## Abstract

M18 aspartyl aminopeptidases (DAPs) are well characterized in microbes and animals with likely functions in peptide processing and vesicle trafficking. In contrast, there is a dearth of knowledge on plant aminopeptidases with a preference for proteins and peptides with N-terminal acidic residues. During evolution of the Plantae, there was an expansion and diversification of the M18 DAPs. After divergence of the ancestral green algae from red and glaucophyte algae, a duplication yielded the *DAP1* and *DAP2* lineages. Subsequently *DAP1* genes were lost in chlorophyte algae. A duplication of *DAP2*-related genes occurred early in green plant evolution. *DAP2* genes were retained in land plants and picoeukaryotic algae and lost in green algae. In contrast, *DAP2*-like genes persisted in picoeukaryotic and green algae, while this lineage was lost in land plants. Consistent with this evolutionary path, *Arabidopsis thaliana* has two *DAP* gene lineages (*AtDAP1* and *AtDAP2)*. Similar to animal and yeast DAPs, AtDAP1 is localized to the cytosol or vacuole; while AtDAP2 harbors an N-terminal transit peptide and is chloroplast localized. His_6_-DAP1 and His_6_-DAP2 expressed in *Escherichia coli* were enzymatically active and dodecameric with masses exceeding 600 kDa. His_6_-DAP1 and His_6_-DAP2 preferentially hydrolyzed Asp-*p*-nitroanilide and Glu-*p*-nitroanilide. AtDAPs are highly conserved metallopeptidases activated by MnCl_2_ and inhibited by ZnCl_2_ and divalent ion chelators. The protease inhibitor PMSF inhibited and DTT stimulated both His_6_-DAP1 and His_6_-DAP2 activities suggesting a role for thiols in the AtDAP catalytic mechanism. The enzymes had distinct pH and temperature optima, as well as distinct kinetic parameters. Both enzymes had high catalytic efficiencies (*k*_cat_/*K*_m_) exceeding 1.0 x 10^7^ M^-1^ sec^-1^. Using established molecular chaperone assays, AtDAP1 and AtDAP2 prevented thermal denaturation. AtDAP1 also prevented protein aggregation and promoted protein refolding. Collectively, these data indicate that plant DAPs have a complex evolutionary history and have evolved new biochemical features that may enable their role in vivo.

## Introduction

The complement of aminopeptidases that preferentially cleave N-terminal aspartyl residues from protein and peptide substrates have been characterized in prokaryotes, animals and yeast. There has been a rigorous genetic dissection and biochemical characterization of the cohort of aspartyl aminopeptidases in *Salmonella typhimurium*. These enzymes are members of four peptidase families: M17 peptidase (PepB), GAT-1 hydrolase (PepE), isoaspartyl dipeptidase (IadA and IaaA), and M20B peptidase (DapE) families [1–4]. More recently, a microbial M18 peptidase capable of cleaving both Glu and Asp residues from chromogenic substrates was identified and crystallized [5].

While the mammalian aminopeptidases that hydrolyze Asp residues have not undergone the rigorous genetic dissection of the γ-proteobacteria, these aminopeptidases are of considerable interest due to their regulatory roles in the renin-angiotensin system that controls blood pressure homeostasis [6, 7]. To date, two enzymes that hydrolyze acidic residues are well characterized in animals. The membrane-bound glutamyl aminopeptidase, also known as aminopeptidase A or ENPEP (EC 3.4.11.7), is a member of the M1 metallopeptidase family. This Ca^2+^-stimulated enzyme hydrolyzes both Glu- and Asp-β-naphthylamide substrates, with a 8.1-fold preference for Glu [8]. This enzyme converts the bioactive peptide angiotensin II to angiotensin III, which controls vasopressin release and blood pressure homeostasis [6, 7].

The second class of eukaryotic aspartyl aminopeptidases (DAPs, DNPEPs, EC 3.4.11.21) are members of the M18 metallopeptidase family (MH clan) and have been characterized in mammals, *Plasmodium falciparum*, *Caenorhabditis elegans*, and yeast [9–13]. The yeast Ape4 (YHR113w), rodent and rabbit brain DAPs^3^, recombinant human His_6_-DAP, as well as the *P*. *aeruginosa* enzyme (PaAP) hydrolyze peptides with N-terminal Asp and Glu residues. Their substrates include dipeptides, tripeptides and larger peptides such as angiotensin I and II [5, 10, 12–16]. However, the human, rodent and rabbit DAPs and the yeast Ape4 inefficiently cleave chromogenic or fluorometric substrates, respectively [12, 13, 16]. This feature distinguishes these enzymes from the dog kidney DAP that can hydrolyze both Asp- and Glu-β-naphthylamide substrates [9], and the *C*. *elegans* DNPP, *P*. *falciparum* PfM18AAP and *P*. *aeruginosa* AP that readily hydrolyze Glu- and Asp-fluorogenic and -*p*-nitroanilide substrates [5, 11, 17].

All eukaryotic M18 DAPs are multimeric. Initial reports indicated that the mammalian and *P*. *falciparum* DAP subunits assembled into octamers [12, 18]. In contrast, recent X-ray crystal studies indicate that similar to the yeast Ape4, the human, *P*. *falciparum*, bovine, and *P*. *aeruginosa* DAPs are dodecameric with tetrahedron-like structures [5, 14, 16, 17, 19]. The X-ray crystal structures have identified the residues that facilitate the coordination of the divalent cations in each subunit, interdigitate with neighboring subunits, line the catalytic pocket, and are required for catalysis [14, 16, 17, 19]. In addition, His to Phe substitutions in the human DAP (DNPEP) showed that eight conserved His residues are important for DAP structure and/or function [13]. Ala substitutions at His401, Asp236 and His82 showed the importance of these residues in coordination of divalent cations and catalysis [5].

To date there are only two reports of plant aminopeptidases that cleave acidic residues. The *Arabidopsis thaliana* asparaginase 1 (At5g08100) has isoaspartyl dipeptidase activity [20, 21]. In addition, a multimeric aminopeptidase that hydrolyzes Glu and Asp residues from peptides and β-naphthylamide substrates was identified in soybean cotyledons [22]. The soybean aminopeptidase is three fold more active on substrates with N-terminal Glu residues. In addition, *in silico* analyses revealed that the *Arabidopsis* genome encodes two proteins (AtDAP1 and AtDAP2) that are highly related to the M18 DAPs [13, 23].

This study characterizes the evolution, location and biochemical characteristics of the understudied chlorophyte DAPs. We show that DAPs are highly conserved proteins in plants, mosses and algae, and, unlike other eukaryotes, most green plants have an expanded repertoire of DAPs with unique subcellular localizations. The expression programs of *AtDAP1* and *AtDAP2* indicate both RNAs and proteins are ubiquitous. AtDAP1 and AtDAP2 are dodecameric enzymes and have biochemical features that distinguish them from each other and previously characterized M18 DAPs. Based on three independent assays, AtDAP1 is bifunctional with both aspartyl aminopeptidase and molecular chaperone activities. In contrast, AtDAP2’s chaperone activity is less robust only being revealed in one of three chaperone assays.

## Materials and methods

### Identification of plant, moss and green algal DAPs

*AtDAP1* (At5g60160) and *AtDAP2* (At5g04710) were identified by protein sequence identity with the human DAP (DNPEP) [13, 23]. Gymnosperm, monocot, eudicot, *Physcomitrella patens* (moss), *Selaginella moellendorffii* (club moss), and green algae (*Chlamydomonas reinhardtii*, *Micromonas* spp., and *Ostreococcus* spp.), oomycete (*Phytothphora infestans*), fungal (*Aspergillus oryzea*) DAPs were identified by BLASTP interrogation of the non-redundant protein database using *AtDAP1* and *AtDAP2* protein sequences (S3 Table). When truncated DAP proteins were identified, expressed sequence tag databases were searched using TBLASTN to assemble full-length coding regions. Signal P, ChloroP, TargetP, Predator, and Plant-mPLoc were used to identify the presence or absence of N-terminal targeting sequences [24–27]. TargetP and ChloroP were used to predict the locations of transit peptide cleavage sites (S3 Table). NCBI’s BLASTP suite was used to identify known conserved domains in chlorophyte DAPs. Splice sites were determined using TAIR coordinates for *Arabidopsis DAPs* and by comparisons of mRNA and genomic DNA sequences; POGS (Putative Orthologous Groups Database) was used to determine rice *DAP* gene splice sites [28]. The location of splice sites relative to the protein sequences were determined by alignments of nucleotide (not shown) and protein sequences using the Multiple Sequence Alignment tool in TCoffee [29].

### Phylogenetic analyses

Multiple sequence alignments were constructed using TCoffee [29] and ProbCons [30] to build progressive pairwise alignments, which enabled assembly of some chlorophyte DAP protein sequences, when full-length clones or genomic regions were not available (S3 Table). Alignments were trimmed with trimal 1.4 using the–automated1 parameter [31]. These alignment and quality steps produced an alignment with 388 informative characters. Phylogenetic trees were prepared using Maximum Likelihood (IQ-TREE 1.5.4) [32] with the best substitution model selected as LG+I+G4 by ModelFinder. Likelihood confidence in the node relationships was generated from 1000 bootstrap replicates using the IQ-TREE ultrafast bootstrap parameters and SH-aLRT test (parameters: -m MFP -bb 10000 -alrt 1000) [32]. Hypothesis testing for placement of lineages within the phylogenetic tree in order to assess likely location for the green algae *DAP1*-like gene used RAxML SH-Test option to evaluate the alternative topologies [33].

### cDNA cloning and construction of His_6_-DAP fusions

Total RNA was isolated from 1-week-old seedlings of *A*. *thaliana* ecotype Columbia by the hot-phenol method [34]. First-strand cDNA was synthesized with total leaf RNA (5 μg) and oligo (dT) primers using the Smart PCR cDNA synthesis kit (Clontech, Palo Alto, CA). Primers used for PCR amplification of the *AtDAP1* (At5g60160) and *AtDAP2* (At5g04710) cDNAs were designed to include restriction enzyme sites for subsequent cloning. For *AtDAP1*, *Nhe* I and *Eco* RI restriction sites (underlined) were added to the forward (5'-ACGCTAGC*ATG*GATAAGAGCTCCCTC-3') and reverse (5'-GCGAATTCTCAA-ACGTCGATAGTGAG-3') primers, respectively. The *DAP1* translational initiation codon is noted in italics. The primer sets amplified the entire coding region of *DAP1* cDNA (nucleotides 86 to 1519) (accession NM_125409). AtDAP2 has a transit peptide of 63 residues (S3 Table). For this reason, the *DAP2* primers were designed to exclude the AtDAP2 transit peptide and amplify the mature-coding region of the *AtDAP2* cDNA corresponding to nucleotides 129 to 1700 (accession NM_120553); the primers used were the forward (5'-TCAT*ATG*GCGTCGATTGT-TGGGGAT-3') and reverse (5'-GCTCGA-GTTAATCATCCACAACGAGC-3') primers containing *Nde* I and *Xho* I sites, respectively.

After initial denaturation of the gene-specific primers and leaf cDNA at 95°C for 2 min, three PCR cycles of 30 sec at 94°C, 30 sec at 52°C, and 2 min at 72°C were performed. At this time, the annealing temperature was increased to 65°C and continued for 30 more cycles. The PCR products amplified using Ex-Taq (Takara, Madison, WI) were cloned into pGEM T-easy vector (Sigma, St. Louis, MO) to generate pGEM-DAP1 and pGem-DAP2. The cDNAs sequences were verified by DNA sequencing. PGEM-DAP1 and pGEM-DAP2 were digested with *Nhe* I and *Eco* RI or *Nde* I and *Xho* I, respectively, and cloned into the pET28 expression vector (Novagen, Darmstadt, Germany). The resulting clones, pET-DAP1 and pET-DAP2, expressed AtDAP proteins with N-terminal His_6_ fusions (His_6_-DAP1 and His_6_-DAP2).

### Over-expression and purification of His_6_-DAP

pET-DAP1 and pET-DAP2 were transformed into BL21[DE3]pLys competent cells. Cells were cultured overnight in LB with kanamycin (50 μg/ml) at 37°C. The overnight culture (20 ml) was diluted 1:20 in LB with kanamycin (50 μg/ml), grown for 3 hr at 30°C and induced with 1 mM IPTG (isopropyl β-D-1-thiogalactopyranoside). Cells were harvested 5 hr later. Cell pellets were resuspended in 5 ml of Buffer A (50 mM NaH_2_PO_4_, 300 mM NaCl) with 10 mM imidazole (pH 8.0) and 1 mg/ml lysozyme and incubated for 30 min on ice. Cells were lysed on ice using five 30-sec pulses; each pulse was followed with a 30-sec resting interval. Cleared lysates were collected and His-DAP proteins were purified on a 1-ml Ni-NTA (nitrilotriacetic acid) column (Qiagen, Valencia, CA) as described previously [35]. His_6_-DAP proteins were concentrated using a Centricon filter (100-kDa MWCO, Millipore, Bedford, MA) to remove low molecular mass proteins, including small amounts of DAP monomers. During this step, the buffer was changed to phosphate-buffered saline (PBS; 164 mM NaCl, 2.7 mM KCl, 10 mM Na_2_HPO_4_, 1.8 mM KH_2_PO_4_, pH 7.5). His_6_-DAP proteins were quantified using BCA protein assay system (Pierce, Rockford, IL) with bovine serum albumin (BSA) as a standard. Aliquots of His_6_-DAP proteins were supplemented to 40% glycerol and stored at -80°C until use. Both His_6_-DAP proteins accumulated as abundant soluble enzymes with yields of > 20 mg per liter of culture. His_6_-DAP activity is stable for > 2 weeks when stored at -20°C.

### Mass determination of His_6_-DAP1 and His_6_-DAP2

SDS-PAGE and native-PAGE as previously described [36]. The molecular masses of His_6_-DAP1 and His_6_-DAP2 were determined according to the methods described by Hedrick and Smith [37] and Bryan [38]. BSA (66-kDa monomer and 132-kDa dimer) and urease (272-kDa trimer and 545-kDa hexamer) were used as molecular mass standards (Sigma). Gels were stained with Coomassie Brilliant Blue R-250 for 16 hr and destained. The relative mobilities of His_6_-DAP and marker proteins were determined in a series of native gels (4, 4.5, 5, and 6% acrylamide). Molecular masses of the proteins were determined from the plot of retardation coefficients [38].

### Enzyme kinetics and effects of divalent ions

His_6_-DAP enzymes (5 μg) were activated in 50 mM Tris-HCl (pH 8.0) containing 0.5 mM MnCl_2_ at room temperature (RT) for 30 min prior to addition of the substrate Asp-*p*-nitroanilide (Asp-*p*-NA; Bachem, Bubendorf, Switzerland). To determine initial velocity of hydrolysis of Asp-*p*-NA, hydrolysis of Asp-*p*-NA was determined at 410 nm at 15 sec intervals for 3 min in Asp-*p*-NA concentrations ranging from 0.5 to 5 mM. His_6_-DAP specific activity was calculated using the molar extinction coefficient of *p*-NA (8899 M^-1^ cm^-1^) at 410 nm. Experiments were repeated a minimum of three times.

Reactions to assess the ability of divalent ions to activate His_6_-DAP were performed as described above with 5 mM Asp-*p*-NA. His_6_-DAP enzymes were preincubated with divalent cations (0, 0.1 or 0.5 mM) in 50 mM Tris-HCl (pH 8.0) for 30 min at RT. Reactions were initiated with the addition of substrate and terminated after 10 min at 37°C with a half volume of 20% trichloroacetic acid (TCA). The reactions were centrifuged for 5 min in a microcentrifuge and supernatants were recovered. Hydrolyzed *p*-NA was determined spectrophotometrically at 410 nm. Experiments were repeated a minimum of three times.

### pH and temperature dependence of His_6_-DAP

To determine the pH optimum for His_6_-DAP activity, His_6_-DAP enzymes (5 μg) were pre-incubated with 0.5 mM MnCl_2_ in the Ellis and Morrison buffer [39] at room temperature for 30 min prior to addition of the Asp-*p*-NA substrate (5 mM). This buffer system, containing 0.1 M *N*-(2-acetamido)-2-amino ethanesulfonic acid, 0.052 M Tris-HCl and 0.052 mM ethanolamine, was used to maintain pKa value and keep ionic strength constant throughout the pH range tested (pH 6 to 9.5). Reactions were incubated at 37°C for 10 min and activity was determined as described above. Assays were performed in triplicate.

The activity of His_6_-DAP at temperatures ranging from 10°C to 90°C was determined in 10°C intervals. The His_6_-DAP enzymes were preincubated in 0.5 mM Tris-HCl (pH 8.0) with 0.5 mM MnCl_2_ at room temperature for 30 min. The mixture was incubated at the reaction temperature for 2 min prior to the addition of substrate and reaction continued for 10 min. The reaction was terminated and product was quantified as described above.

### Chemical inhibitors

Mn^2+^-activated His_6_-DAP enzymes in 0.5 mM Tris-HCl (pH 8.0) were preincubated with chemicals at room temperature for 30 min. Asp-*p*-NA (5 mM) was added and the reaction transferred to 37°C for 10 min. The reaction was terminated and product was quantified as described above. EDTA, 1,10-phenathroline, bestatin, Antithrombin III, E64, PMSF, and DTT were purchased from Sigma. Aprotinin was purchased from EMB Millipore.

### Substrate specificity

Seventeen amino acyl-*p*-nitroanilide (aa-*p*-NA) substrates were used as substrates for the Mn^2+-^activated His_6_-DAPs. aa-*p*-NAs were purchased from Bachem or Sigma. Due to differences in aa-*p*-NA solubilities, aa-*p*-NA substrate stocks (200 mM) were prepared in ethanol (Asp-, Ile-, Met-, Val-, Ala-, Leu-, Pro-, and Thr-*p*-NA), water (Arg- and Lys-*p*-NA), methanol (Gly-*p*-NA), or dimethylsulfoxide (DMSO; Glu-*p*-NA). The impact of each solvent on His_6_-DAP1 activity was tested in reactions with 1–10 mM Asp-p-NA substrate; His_6_-DAP1 had similar activity in reactions in water or 0.5–5% v/v ethanol, methanol or DMSO. For the substrate specificity studies, solvent levels did not exceed 2.5%. Substrate specific activities were determined spectrophotometrically as described above. Activities were expressed relative to Asp-*p*-NA.

### Molecular chaperone assays

For the molecular chaperone assays, one-liter cultures were grown at 37°C for His_6_-LAP-A and 23°C for His_6_-DAP1 and His_6_-DAP2, induced with IPTG, cleared lysates prepared, and His_6_-tagged proteins purified using Ni-NTA resin columns [35]. His_6_-LAP-A and His_6_-DAPs were concentrated using Amicon Ultra-2 centrifugal filters and protein purity was determined by staining of SDS-PAGE gels.

The ability of 0–2 μM of His_6_-DAP1, His_6_-DAP2, or His_6_-LAP-A (positive control) to protect the restriction enzyme NdeI from thermal inactivation at 43°C was determined according to Scranton et al [35]. NdeI-digestion products were separated on 1% agarose gel stained with ethidium bromide. NdeI digestion of plasmid DNA (cLEX-6-H6) at 37°C (without the 43°C incubation) served as a positive control and produced 4.6-kb and 0.2-kb fragments.

The citrate synthase (CS) aggregation assay was performed as previously described [35]. Briefly, the reactions contained 300 nM citrate synthase (Sigma, St. Louis, MO), 50 mM HEPES-KOH (pH 7.5), 5% glycerol, and purified His_6_-DAP1 (600 nM), His_6_-DAP2 (300 nM), or His_6_-LAP (900 nM). The reaction was placed in a plastic cuvette, heated to 43°C, and light scattering (360 nm) was measured at indicated times (0–60 min) using a NanoDrop 2000c (Thermo Scientific, Rockford, IL). His_6_-DAP2 was used at lower concentrations since this protein tended to spontaneously aggregate at concentrations higher than 300 nM.

The microtiter plated-based luciferase refolding assay was performed according to Scranton et al [35]. Prior to assays, His_6_-DAP1, His_6_-DAP2 and His_6_-LAP-A proteins were dialyzed against Buffer A using “V” series membranes (0.05 μM; Millipore) to remove glycerol and imidazole. QuantiLum Recombinant Luciferase (1 μM; Promega, Madison, WI) in 2.5 mM HEPES–KOH (pH 7.5), 5 mM MgCl_2_, 150 mM KCl, and 2 mM dithiothreitol (DTT) was mixed with 0–6 μM His_6_-DAP1, His_6_-DAP2 or His_6_-LAP. Samples were heated for 11 min at 42°C and chilled on ice for 5 min. Heated samples (1 μl) were added to the reactivation mix (40 μl total volume) that included 24 μl rabbit reticulocyte lysate (RRL; Promega), 25 mM HEPES-KOH (pH 7.5), 2 mM ATP, 5 mM MgCl_2_, 10 mM KCl, and 1 mM DTT. For each reaction, three 10-μl aliquots of the reactivation mix with heated samples were distributed into individual wells of microtiter plates. Plates were incubated at 30°C and luminescence was measured using a LUMIstar Galaxy luminometer (BMG Labtechnologies, Offenberg, Germany) with an integration time of 10 sec. Percent activity corresponds to the relative luminescence compared to unheated luciferase control.

## Results

### Plant DAPs have conserved motifs

The M18 peptidase family includes two related classes of metallopeptidases: the aspartyl aminopeptidases with the M18_DAP motif and aminopeptidase I (API, ApeI)-like proteins with the M18_API motif [12, 16, 18, 40, 41]. API-like enzymes preferentially hydrolyze peptides with hydrophobic N-terminal residues (i.e., Leu, Phe, Ala) and are common in the Ascomycetes (eg., *S*. *cerevisiae* Ape I) [41]. However, interrogation of green plant genomes and ESTs indicated that neither algal or land plant genomes encode M18_API orthologs.

**Fig 1:**
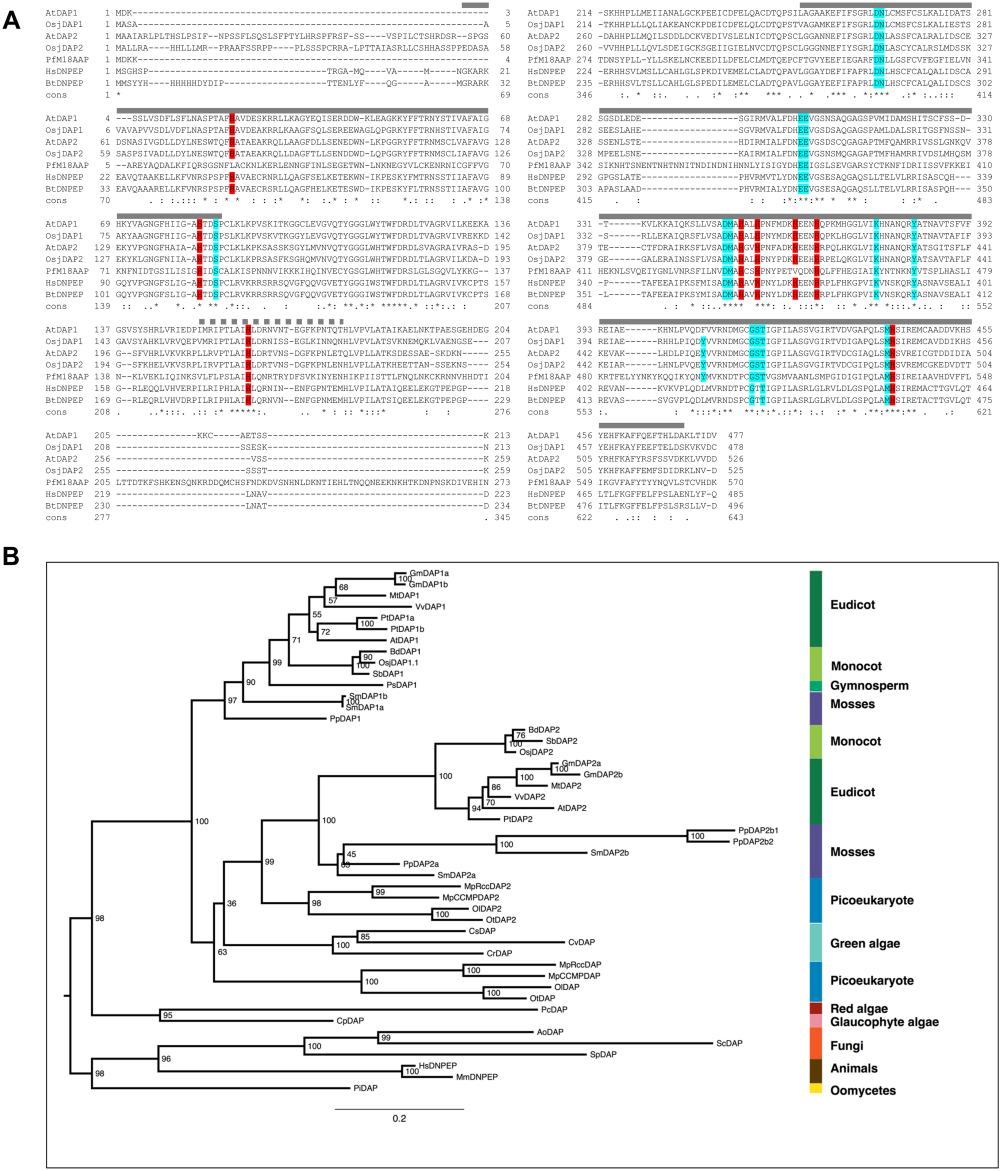
DAP alignments and phylogenetic relationships. *A*, Alignments of the *Arabidopsis* DAP1 (At5g60160) and DAP2 (At5g04710), *Oryza sativa* DAP1.1 (Os12g13390.1) and DAP2 (Os01g73680), *P*. *falciparum* 3D7 M18AAP, *Homo sapiens* DNPEP, and *Bos taurus* DNPEP proteins are shown. Accession numbers are found in S3 Table. The two regions that form the globular proteolytic domain based on X-ray crystal structures of the human DNPEP, bovine DNPEP and *P*. *falciparum* PfM18AAP are indicated by the heavy grey lines above the protein sequences [14, 17, 19]. The dimerization domain is located between the proteolytic domains and includes the flexible loop, which contains the His residue that inserts into the catalytic site of its adjacent subunit (dashed grey line) (S1 Table). His residues that alter DNPEP activity based on biochemical studies and/or X-ray data are shown in red [13, 14, 17, 19]. Residues that have a role in metal coordination, substrate binding/catalysis or that line the catalytic pocket based on one or more X-ray structures are shown in teal. Residues numbers for the human DNPEP, bovine DNPEP and PfM18AAP are for the total protein and differ from residue numbers in the crystal structure determination [19]; these correlations are provided in S1 Table. Conserved amino acids (Cons) are indicated and identical residues in the seven DAPs are indicated with a *. *B*, Phylogenetic relationships of chlorophyte DAPs. Chlorophyte DAPs include DAPs from *Arabidopsis thaliana* (At), *Brachypodium distachyon* (Bd), *Chlamydomonas reinhardtii* (Cr) *Chlorella variabilis* (Cv), *Coccomyxa subellipsoidea* (Cs), *Cyanophora paradoxa* (Cp), *Glycine max* (Gm), *Medicago truncatula* (Mt), *Micromonas pusilla* (Mp), *Oryza sativa* japonica (Osj); *Ostreococcus lucimarinus* (Ol), *O*. *tauri* (Ot), *Physcomitrella patens* (Pp), *Populus trichocarpa* (Pt), *Porphyridium cruentum* (Pc), *Picea sitchensis* (Ps), *Selaginella moellendorfii* (Sm), *Sorghum bicolor* (Sb), and *Vitis vinifera* (Vv). *Homo sapiens* (HsDNPEP), *Mus musculus* (MmDNPEP), *Phytothphora infestans* (PiDAP), *Aspergillus oryzae* (AoDAP), *Saccharomyces pombe* (SpDAP) and *Saccharomyces cerevisiae* (ScApe4; ScDAP) DAPs served as outgroups. Accession numbers for all DAP proteins are found in S3 Table.

In contrast, based on sequence identity with human DNPEP, two *Arabidopsis* DAP-encoding genes were identified [12, 23]. AtDAP1 and AtDAP2 proteins share ~49% amino acid residue identity with the human DAP [13, 19]. AtDAP1 and AtDAP2 have M18_DAP motifs that span residues 13 to 463 and 73 to 512, respectively (Fig 1). Based on X-ray crystal structures for the human DNPEP, bovine DNPEP and *Plasmodium* PfM18AAP, the globular proteolytic domain spans residues 1 to 116 and residues 249 to 472 in AtDAP1 and the dimerization domain is the intervening region (residues 117 to 248) (Fig 1A). The eight His residues that are important for human DNPEP structure and function are conserved (Fig 1A; S1 Table). This includes the AtDAP1’s His161 and AtDAP2’s His218, which correspond to the human, bovine and *Plasmodium* His residues that reside within a flexible loop. This loop interacts with an adjacent DAP subunit to constrain the size of the active site [14, 17, 19]. In addition, with one exception, all residues predicted to be critical for coordination of the two zinc ions, substrate catalysis, and lining of the catalytic pocket identified in recent DAP protein crystal structures are conserved [14, 17, 19] (Fig 1A; S1 Table). The exception is AtDAP1’s Phe406, which is located in the catalytic pocket. This residue is a Tyr in AtDAP2 and *P*. *falciparum*, while it is a Leu in animals (S1 Table).

**Fig 2:**
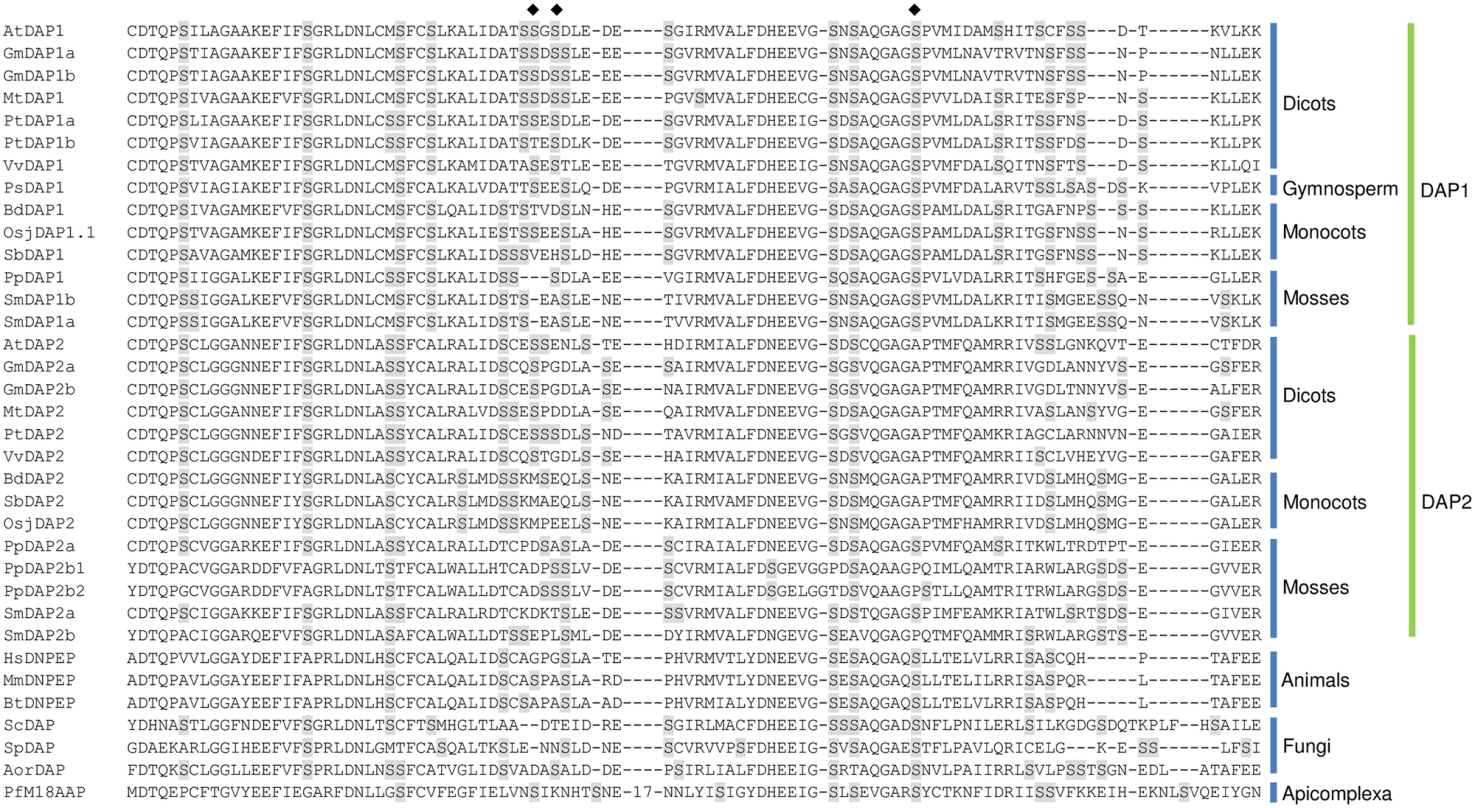
A phosphorylated Ser residue AtDAP1 is imbedded in a Ser-rich region. The DAP1s and DAP2s from plants and moss, and DAPs from fungi, animals and *Plasmodium* were aligned by T-coffee and the region from AtDAP1 residues 243 to 336 is displayed. Ser residues are highlighted in gray. Ser284 is phosphorylated in *AtDAP1* and two predicted phosphorylation sites Ser282 and Ser312 are also shown (♦). AtDAP1’s Thr194 is also predicted as a phosphorylation site (region is not shown). The *Plasmodium* PfM18AAP has a 17-residue insertion (NTNHTNNITNDINDNIH) in this region.

The Musite program predicts that several Ser and/or Thr residues may be phosphorylated in plant DAPs (S2 Table) [42]. The majority of the predicted Ser/Thr phosphorylation sites within DAPs reside within the C-terminal portion of the bipartite proteolytic domain (S2 Table; Fig 1A). While four potential phosphorylation sites are predicted for AtDAP1 (eg., Thr194, Ser282, Ser284, and Ser313) [42], mass spectrometry analyses of proteins from *Arabidopsis* Col and Ler cell cultures indicate that only AtDAP1’s Ser284 is phosphorylated [43, 44]. Ser284 is imbedded in a Ser-rich region of DAP1s. Plant and moss DAP1 proteins have ~8.4% Ser overall; while, the C-terminal proteolytic domain region surrounding Ser284 is significantly higher in Ser content (~16.1% Ser) (Fig 2). In contrast, plant and moss mature DAP2s and fungal DAPs have a mean Ser content of 9.1%, and the Ser284 region was modestly enriched in Ser residues (11.6%) (Fig 2; S2 Table). The animal DNPEPs and *Plasmodium* M18AAP have lower Ser contents when the total protein and the proteolytic domain were compared (7.7 vs 9.1%).

### Expansion of the M18_DAP protein family in the chlorophyte lineage

Surprisingly, AtDAP1 and AtDAP2 shared only 59% amino acid residue identity suggesting an ancient duplication event gave rise to the two genes. To assess the time of the *DAP1* and *DAP2* duplication relative to key events in land plant evolution, DAPs from six dicots, five monocots, one gymnosperm, a moss, a clubmoss, and eleven algae were identified (S3 Table). For context, green plants (green algae and land plants), glaucophyte algae and red algae are monophyletic deriving from an ancestral first algae [45]. The flagellated ancestral algae that defined the green plant lineage split from the glaucophyte and red algae lineages approximately 1500 Mya [46, 47]. Between 425–700 Mya, the Streptophyta and Chlorophyta diverged [46, 48]. The Streptophyta gave rise to vascular and non-vascular land plants and some green algae. The Chlorophyta includes the prasinophytes and the core chlorophyte algae (eg., *Chlorella*, *Cocomyxa*, and *Chlamydomonas)*. Prasinophytes are more primitive than the core chlorophyte algae and include the picoeukaryotic algae of the Mamiellales (eg., *Ostreococcus lucimarinus*, *O*. *tauri*, and *Micromonas pusilla*). The core chlorophyte algae adapted to fresh water environments [49].

To determine the evolution of *DAP* genes in the Plantae, two mammalian (human and mouse), an oomycete (*Phytothphora infestans*) and three fungal (*Aspergillus oryzae*, *Saccharomyces cerevisiae*, and *Schizosaccharomyces pombe*) DAPs were used as out-groups to construct maximum likelihood (IQ-TREE) phylogenetic trees. While single genes encoding proteins with the hallmark M18_DAP motif are found in animals, *Plasmodium*, oomycetes, and fungi [11, 12, 16, 18], the DAP protein family is expanded in most chlorophytes (Fig 1B; S3 Table). There is strong evidence for two *DAP* lineages (*DAP1* and *DAP2*) in land plants and maximum likelihood phylogeny provides evidence for additional *DAP* duplications and paralog loss within green plant evolution.

A single *DAP* gene is found in the glaucophyte (*Cyanophora paradoxa*) and red alga (*Porphyridium cruentum*). While all land plants have *DAP1* genes, no *DAP1* genes were identified in green picoeukaryotic algae or green algae. These data suggest a duplication of *DAP* genes occurred after the divergence of the green plant ancestor from the glaucophyte and red algae. *DAP1* genes were subsequently lost from green algae and picoeukaryotic algae after the divergence of the land plants. Furthermore, it suggests that *DAP1* genes are not essential for unicellular green algae.

In contrast, the prasinophyte genomes (eg. *Ostreococcus spp*. and *M*. *pusilla*) support the premise of a *DAP* duplication early in green plant evolution. This is evidenced by the strongly supported sister relationship of chlorophyte and prasinophyte *DAP2* gene lineage and a more primitive *DAP* gene branch. The primitive *DAP* genes were found in green picoeukaryotic algae; this gene lineage was lost in land plants.

Three algal species that represented the core fresh water chlorophytes were evaluated. This included the two members of the Trebouxiophyceae (*Chlorella variabilis* and *Cocomyxa subellipsoidea)* and one member of the Chlorophyceae *(Chlamydomonas reinhardtii*). All three algae have a single *DAP* gene, which appears as a sister clade to the *DAP2* genes. Collectively these data support the notion that only one *DAP* gene is needed in Chlorophytes (Fig 1B; S3 Table).

Further expansions of the *DAP1* and/or *DAP2* gene families occurred in land plants including the bryophytes (moss; *Physcomitrella patens*) and lycopodiophytes (clubmoss; *Selaginella moellendorffii*), as well as some angiosperms. The bifurcations of the *DAP1* and *DAP2* trees were consistent with the current general perceptions of Viridiplantae evolution [50]. The branches for the moss, club moss and seed plants, as well as the eudicot and monocot branches, were well supported (Fig 1B). The expansions in the *DAP2* gene family occurred in primitive plants (i.e., before *P*. *patens* and *S*. *moellendorffii* emerged) after seed plant divergence (Fig 1B; S3 Table). Further duplications are relatively recent and independent as the moss and clubmoss genomes each have two *DAP2* paralogs. In addition, *P*. *patens* harbors an even more recent duplication event that created *PpDAP2b1* and *PpDAP2b2* paralogs, which share 87% amino acid identity (Fig 1B; S3 Table).

The *DAP2* gene family expansion was not evidenced in any of the monocots [rice (*Oryza sativa japonica*), sorghum *(Sorghum bicolor*), maize *(Zea mays*), barley (*Hordeum vulgare*), and purple false brome *(Brachypodium distachyon*)] or eudicots [*Arabidopsis thaliana*, *A*. *lyrata*, *Glycine max (soybean)*, *Medicago truncatula* (barrel medic), *Vitus vinifera* (common grape), and *Populus trichocarpa* (black cottonwood)] examined (Fig 1B; S3 Table) Only the soybean genome harbors two *DAP2* genes (*GmDAP2a*, *GmDAP2b*) reflecting the tetraploid nature of its genome [51]. Collectively, these data indicated that the *DAP2* family expansion occurred in an ancestor of the moss and club moss lineage (Fig 1B; S3 Table).

In contrast, land plant *DAP1* genes have evolved in a distinct manner. While the moss genome encodes a single *DAP1* gene (*PpDAP1*), the club moss genome contains two recently duplicated genes (*SmDAP1a* and *SmDAP1b*) encoding proteins with over 94% amino acid residue identity. All of the monocots and most of the eudicot (*Arabidopsis spp*., *M*. *truncatula* and *V*. *vinifera*) genomes examined harbor a single *DAP1* gene (Fig 1B; S3 Table). The exceptions are soybean and *P*. *trichocarpa*. The two soybean *DAP1* genes are consistent with *G*. *max*’s tetraploid origins [51]. In contrast, a recent duplication gave rise to the *Populus DAP1* paralogs (*PtDAP1a and PtDAP1b*) that share 89.6% protein sequence identity.

**Fig 3:**
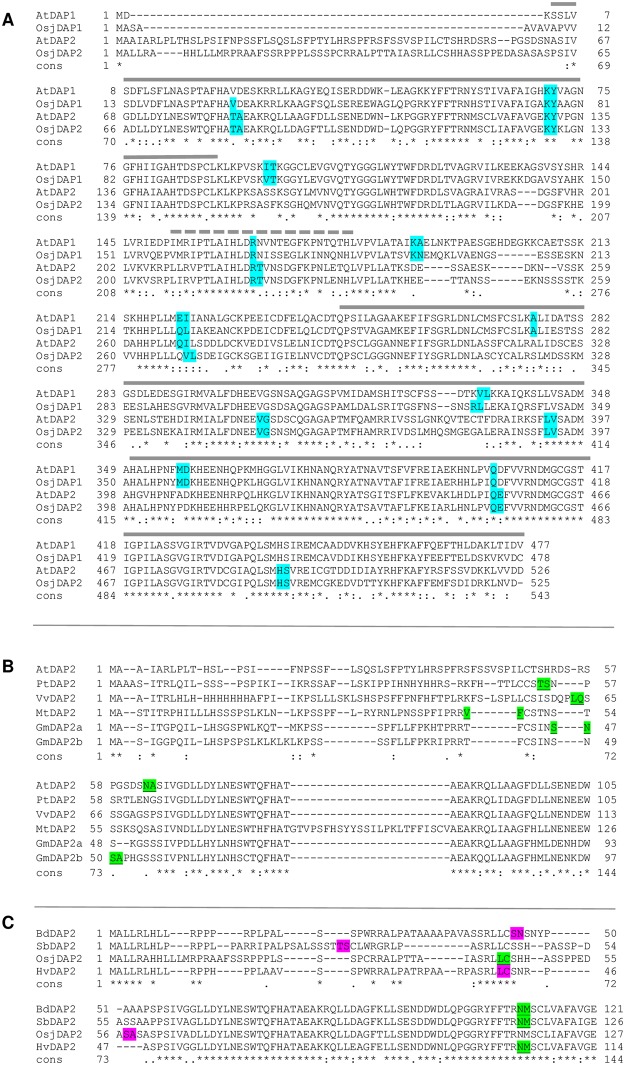
Splice sites and transit peptides of monocot and dicot DAPs. *A*, The location of splice sites in the *Arabidopsis* and rice DAPs to relative to each triplet codon was determined. The location of the splice sites relative to DAP amino acid residues is highlighted in teal. Conserved (cons) amino acid residues are shown and identical residues in all four DAPs are indicated with a *. *B*, T-Coffee multiple sequence alignments of dicot DAP2 N-terminal regions including *A*. *thaliana* (AtDAP2), *P*. *trichocarpa* (PtDAP2), *V*. *vinifera* (VvDAP2), *M*. *truncatula* (MtDAP2), and *G*. *max* (GmDAP2a, GmDAP2b) proteins. TargetP-predicted transit peptide cleavage sites are highlighted in green. In most cases, TargetP and ChloroP predicted sites were identical or in close proximity (S3 Table). *C*, T-Coffee multiple sequence alignments of monocot DAP2 N-terminal regions including *B*. *distachyon* (BdDAP2), *S*. *bicolor* (SbDAP2), *O*. *sativa* (OsDAP2), and *H*. *vulgare* (HvDPA2) proteins. Sequence accession numbers and lengths of predicted transit peptides are provided in S3 Table. TargetP-predicted transit peptide N-cleavage sites are highlighted in green and underlined; ChloroP predictions for transit peptide cleavage sites were different than those predicted by TargetP and are highlighted in magenta.

The ancient duplication that gave rise to the *DAP1* and *DAP2* genes in plants is also evidenced when the architecture of the monocot and dicot *DAP1* and *DAP2* genes are compared. *AtDAP1* and *AtDAP2* have complex structures, spanning greater than 2.8 kb with 11 and 9 exons, respectively. Not only were the numbers of introns different, the relative locations of introns in the *AtDAP1* and *AtDAP2* genes to DAP protein sequences were distinct supporting an ancient evolutionary origin (Fig 3A). Similarly, the rice *DAP1* and *DAP2* gene structures were distinct but when compared to the *Arabidopsis* gene structures several compelling similarities were revealed. First, the rice and *Arabidopsis DAP1* and *DAP2* orthologs had similar numbers of exons/introns. Second, although the rice introns were ≥2.5-fold longer than the *Arabidopsis* introns, the positions of rice and *Arabidopsis DAP1* and *DAP2* introns relative to the coding sequences were conserved (Fig 3A). These data corroborate the phylogenetic analyses suggesting that the divergence of the *DAP1* and *DAP2* lineages preceded monocot/dicot divergence.

### Plant DAPs reside in distinct subcellular compartments

Alignments of monocot and eudicot DAP1 and DAP2 proteins demonstrated that DAP2 proteins have N-terminal extensions relative to DAP1 proteins (Figs 1A and 3A; S3 Table). Target P and Predator predict that the N-terminal transit peptides target the eudicot DAP2s to plastids. This conclusion is strongly supported by the detection of DAP2 within Arabidopsis plastids by LC-MS/MS analyses [52]. In contrast, TargetP, Predator, and Plant-mPLoc predict that the monocot DAP2s reside within the mitochondrion or may have a dual organellar location (S3 Table). Alignments of the plant DAP2 protein N-terminal regions indicated that the monocot DAP2 transit peptides are more conserved than the transit peptides in the eudicot DAP2s (Fig 3B and 3C). These data suggest that the monocot and eudicot DAP2 proteins may have acquired their transit peptides independently or that these N-terminal regions are under different amounts of selective pressure in monocots and eudicots.

The SignalP, Predator, Target P, and ChloroP algorithms predict that plant DAP1 proteins are cytosolic, as they do not contain motifs for targeting to plastids, mitochondria or the endomembrane system. Two proteomics studies have confidently reported AtDAP1 in vacuolar preparations [53, 54].

### Biochemical characterization of AtDAP1 and AtDAP2

**Fig 4:**
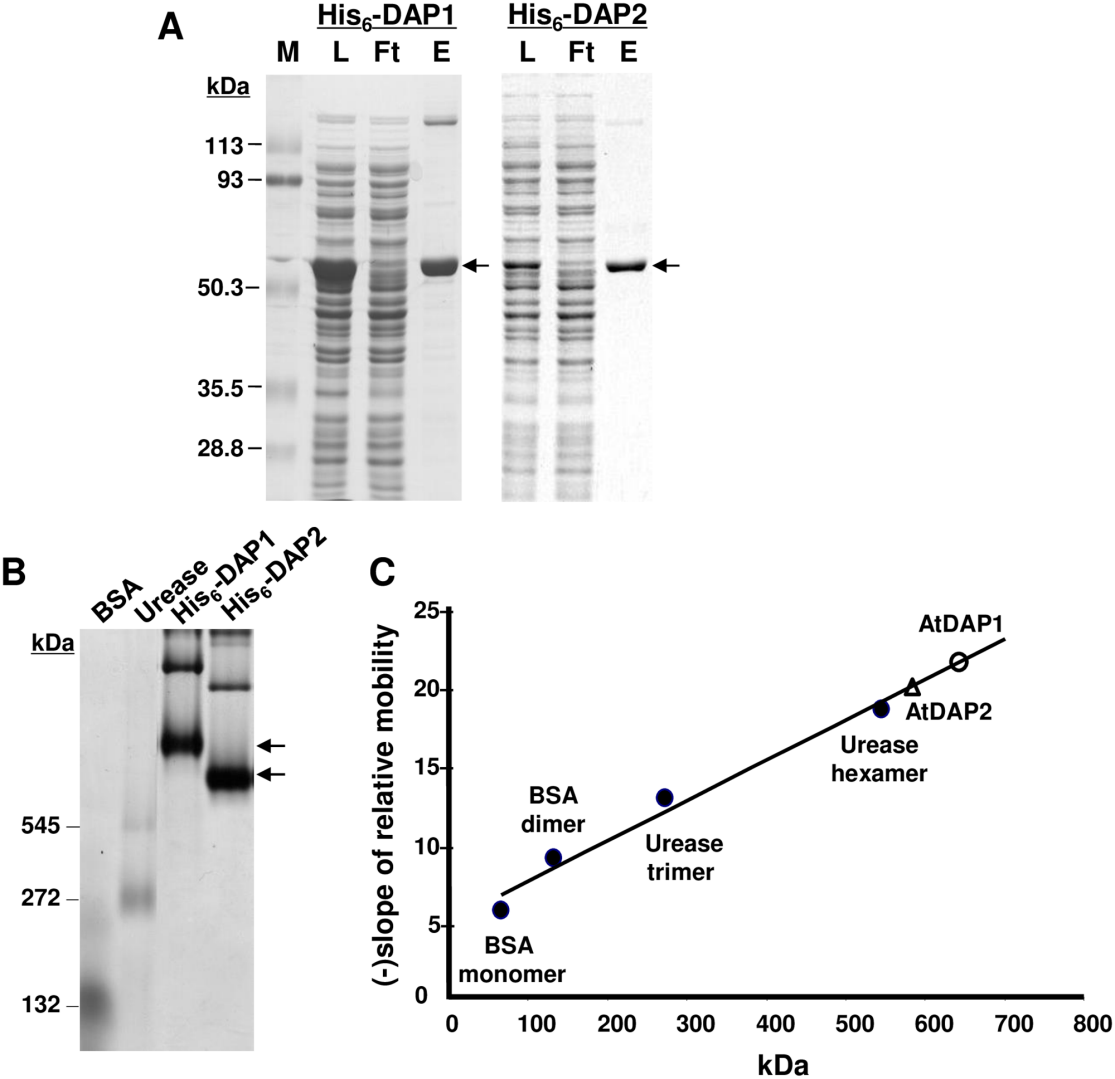
His_6_-DAP1 and His_6_-DAP2 purification, molecular mass and quaternary structure. *A*, Purification of His_6_-DAPs. His_6_-DAP proteins were isolated from IPTG-induced *E*. *coli* cultures, purified by a Ni-NTA chromatography and separated by SDS-PAGE. Lanes represent: protein standard markers in kDa (M), total proteins from a cleared lysate of *E*. *coli* (L), flow through of Ni-NTA column (Ft), proteins eluted from Ni-NTA column by 100 mM imidazole and size selected using a 10-kDa centricon filter (E, eluted). Arrows indicate His_6_-DAP1 and His_6_-DAP2 monomers. *B*, Native PAGE (5%) fractionation of Ni-NTA affinity column-purified of His_6_-DAP1 (20 μg), His_6_-DAP2 (20 μg), as well as BSA (monomer and dimers) and urease (trimer and hexamers) as mass standards. *C*, The relative mobilities of standards and His_6_-DAPs were determined with a series of native gels ranging from 4% to 6% acrylamide. Molecular masses (kDa) were calculated using protein relative mobilities and the retardation coefficient (Bryan 1977).

AtDAP1 and the AtDAP2 protein lacking its 63-residue N-terminal transit peptide (mature AtDAP2) were expressed as His_6_-fusion proteins in *E*. *coli* (His_6_-DAP1 and His_6_-DAP2, respectively). His_6_-DAP1 (54.6 kDa) and His_6_-DAP2 (53.2 kDa) were purified to near homogeneity by Ni-NTA columns (Fig 4A). A faint 120-kDa protein was routinely detected in the purified His_6_-DAP1 preparations. Immunoblot analysis indicated the 120-kDa band contained His_6_-DAP1 and is likely to represent a DAP dimer (data not shown). DAP dimers have been detected for the *Aspergillus* DAP, *Plasmodium* M18APP, and mammalian DNPEP [17, 19, 55]. Dimers are known to be an assembly intermediate for the human and *Plasmodium* dodecamer [17, 19].

The molecular masses of the DAP1 and DAP2 complexes were determined from the relative mobilities of purified His_6_-DAPs using a series of native polyacrylamide gels (Fig 4B). The calculated molecular masses of His_6_-DAP1 and His_6_-DAP2 were 637 and 600 kDa, respectively (Fig 4C). These masses were consistent with the *Arabidopsis* His_6_-DAP enzymes assembling into dodecameric complexes [13, 14, 17, 19].

### His_6_-DAP substrates, cation dependence and inhibitors

The *Arabidopsis* His_6_-DAP1 and His_6_-DAP2 readily hydrolyzed the chromogenic substrate Asp-*p*-nitroanilide. Both His_6_-DAP1 and His_6_-DAP2 had maximal activity in the presence of 0.5 mM Mn^2+^ (Table 1). In the absence of divalent cations or in presence of Mg^2+^ or Ca^2+^, His_6_-DAP1 had less than ~12% of its maximal activity. Unlike His_6_-DAP1, His_6_-DAP2 had substantial activity (~37%) in the absence of added divalent cations, which increased to >51% in the presence of Mg^2+^ and Ca^2+^. In contrast, Zn^2+^ completely inhibited His_6_-DAP1 and His_6_-DAP2 (Table 1). Consistent with its activation by Mn^2+^ ions, EDTA inhibited both His_6_-DAP1 and His_6_-DAP2 activities by 95% and 86%, respectively (Table 2). The chelator 1,10-phenanthroline inhibited His_6_-DAP activities less effectively, with His_6_-DAP1 and His_6_-DAP2 displaying 29% and 42% activity, respectively.

**Table 1:** The effect of divalent cations on His_6_-DAP1 and His_6_-DAP2 activity.

Divalent Cation	Concentration	DAP Activity (%)^A^	
		His_6_-DAP1	His_6_-DAP2
--	0 mM	12.71 ± 0.10	37.13 ± 0.90
MnCl_2_	0.1 mM	76.16 ± 1.60	78.70 ± 0.24
	0.5 mM	100%	100%
MgCl_2_	0.5 mM	11.65 ± 0.32	52.15 ± 0.50
CaCl_2_	0.5 mM	10.50 ± 0.04	50.86 ± 2.60
ZnCl_2_	0.5 mM	<0.5	<0.5

^A^ His_6_-DAPs were incubated in 50 mM Tris-HCl (8.0) in the presence or absence of a divalent cation for 30 min prior to the addition of 5 mM Asp-*p*-NA. Reactions proceeded for 10 min at 37°C, were terminated and hydrolysis was measured spectrophotometrically. Results are expressed as % of maximal His_6_-DAP1 or His_6_-DAP2 activity, which occurred in the presence of 0.5 mM MnCl_2_

**Table 2:** His_6_-DAP1 and His_6_-DAP2 activities after treatment with peptidase inhibitors.

Treatment		% His_6_-DAP Activity^A^	
Chemical	Concentration	His_6_-DAP1	His_6_-DAP2
EDTA	1 mM	6.22 ± 0.15	13.60 ± 0.61
	5 mM	4.88 ± 0.12	13.86 ± 0.37
1,10-phenanthroline	1 mM	61.10 ± 1.40	75.82 ± 0.35
	5 mM	29.27 ± 0.11	42.23 ± 0.62
Bestatin	0.5 mM	93.29 ± 1.40	111.36 ± 1.60
	1.0 mM	89.36 ± 0.32	110.98 ± 1.05
PMSF	1 mM	16.34 ± 0.55	23.52 ± 0.80
	5 mM	7.20 ± 0.48	10.18 ± 1.61
DTT	5 mM	240.20 ± 32.00	238.53 ± 34.52
DTT + 5 mM PMSF	5 mM	10.6 ± 0.36	15.25 ± 1.63
Antithrombin III	1 U/ml	61.30 ± 5.12	38.10 ± 0.17
	2.5 U/ml	13.87 ± 4.37	20.17 ± 3.32
Aprotinin	5 μM	95.00 ± 8.82	88.60 ± 2.85
	20 μM	93.12 ± 2.53	60.70 ± 1.00
E64	0.5 mM	91.25 ± 5.55	108.04 ± 1.03
	1 mM	87.15 ± 5.27	111.61 ± 7.93

^A^ Mn^2+^-activated His_6_-DAPs were incubated with chemicals at the indicated concentrations for 30 min prior to addition of Asp-*p*-NA (5 mM) as described in *Experimental Procedures*. Hydrolysis was measured spectrophotometrically. The experiment was repeated three times and standard deviations are shown. Activities are expressed relative to Mn^2+^-activated His_6_-DAPs activities in the absence of chemicals (100%).

Neither the broad-spectrum aminopeptidase inhibitor bestatin nor the serine protease inhibitor aprotinin inhibited His_6_-DAP1 nor His_6_-DAP2 activity (Table 2). In contrast, both PMSF (a Ser and Cys proteinase inhibitor) and antithrombin III (a Ser protease inhibitor) reduced His_6_-DAP1 and His_6_-DAP2 activities. While the thiol protease inhibitor E64 (1 mM) did not inhibit His_6_-DAP1 and His_6_-DAP2, the reducing agent dithiothreitol (DTT), which stimulates some thiol proteases, increased His_6_-DAP1 and His_6_-DAP2 activity by approximately 2.5 fold. The DTT activation of His_6_-DAPs was inhibited by the addition of 5 mM PMSF. While PMSF is an inhibitor of Ser proteases, it also inhibits the thiol protease papain. DTT counteracts PMSF’s inhibition of thiol proteases. These data suggest that an active site thiol is important for AtDAP1 and AtDAP2 activity.

### Temperature and pH dependence

**Fig 5:**
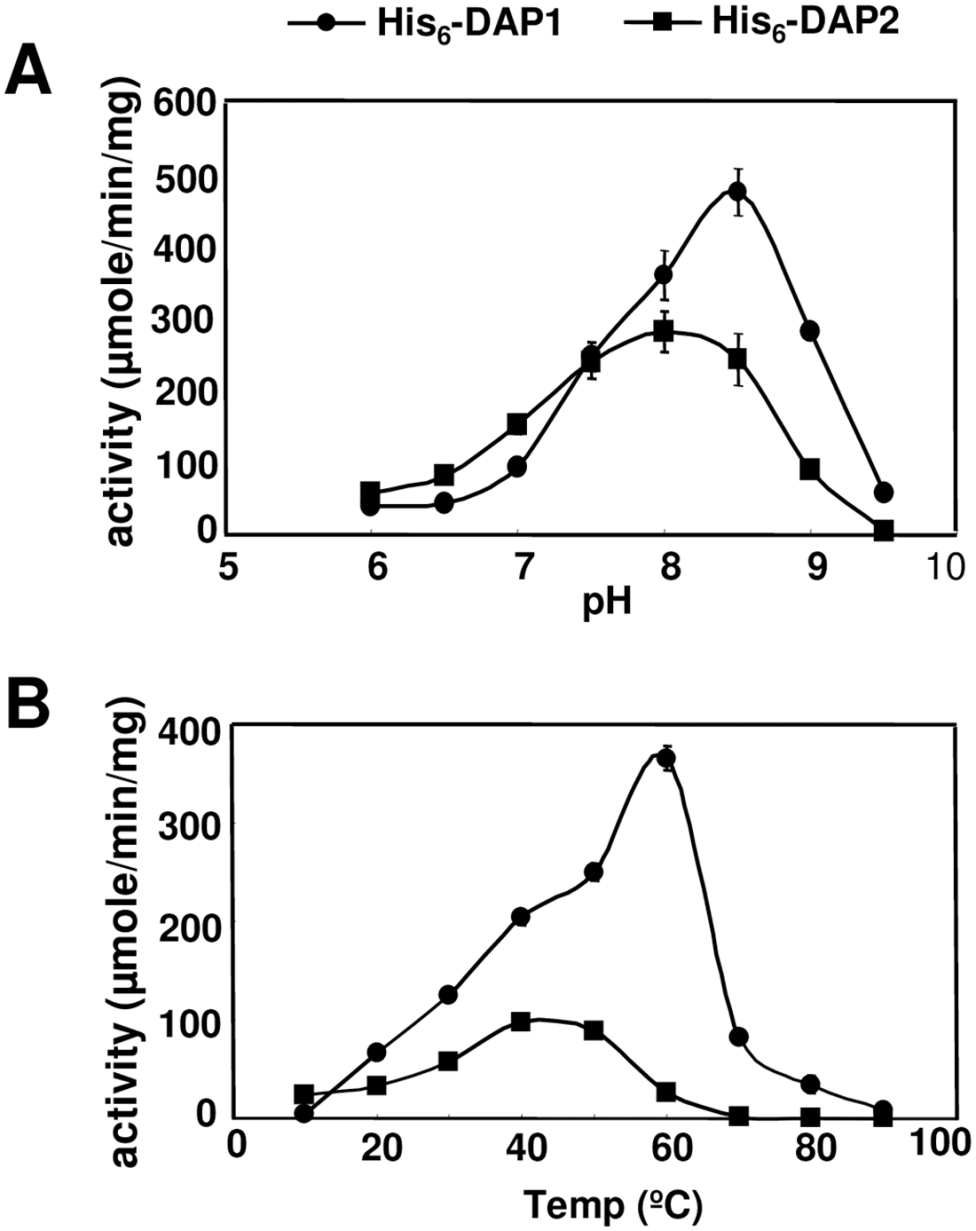
Biochemical characterization of His_6_-DAP1 and His_6_-DAP2. *A*, The effects of pH on the activities of His_6_-DAP1 (●) and His_6_-DAP2 (■) were determined using the Ellis and Morrison buffer system at 37°C using Asp-*p*NA as a substrate. *B*, The optimum temperatures for the activities of His_6_-DAP1 (●) and His_6_-DAP2 (■) were determined at 10°C intervals in the range of 10–90°C using Asp-*p*NA as a substrate at pH 8.0.

Optimum pH for the His_6_-DAPs activity was determined using the multicomponent buffer system of Ellis and Morrison [39] (Fig 5A). Using Asp-*p*-NA as a substrate, the Mn^2+^-activated His_6_-DAP1 and His_6_-DAP2 were maximally active under basic reaction conditions. His_6_-DAP1 had maximum activity at pH 8.5, with a 5-fold increase in activity relative to reactions at pH 7.0. In contrast, His_6_-DAP2's activity was increased only 2 fold at pH 8. The temperature dependence of His_6_-DAP enzymatic activity was tested using the Mn^2+^-activated His_6_-DAP1 and His_6_-DAP2, Asp-*p*-NA as a substrate and Tris-buffered (pH 8) reaction conditions. His_6_-DAP1 was a heat-stable enzyme that had optimal activity at 60°C. In contrast, His_6_-DAP2’s temperature optimum was 40°C and it was inactive at 60°C (Fig 5B).

### Substrate specificity and enzyme kinetics

The substrate specificities of the Mn^2+^-activated His_6_-DAP1 and His_6_-DAP2 enzymes were determined using twelve amino acyl-*p*-nitroanilide (*p*-NA) substrates containing acidic, basic, polar, as well as bulky or hydrophobic residues (Table 3). Both His_6_-DAP1 and His_6_-DAP2 preferentially hydrolyzed substrates with N-terminal acidic residues with rates of hydrolysis of Asp-*p*-NA 2.2- and 1.5-fold greater than Glu-*p*-NA, respectively. His_6_-DAP1 and His_6_-DAP2 hydrolyzed all other amino acyl-*p*-NA substrates at substantially lower rates ranging from 0.1 to 6% of the Asp-*p*-NA rate of hydrolysis.

**Table 3:** Substrate specificities of His_6_-DAP1 and His_6_-DAP2.

	Activity (%)^A^	
Substrate	His_6_-DAP1	His_6_-DAP2
Asp-*p*NA	100	100
Glu-*p*NA	44.65 ± 5.30	62.29 ± 1.77
Arg-*p*NA	< 0.1	1.10 ± 0.57
Lys-*p*NA	0.19 ± 0.33	< 0.1
Ile-*p*NA	2.12 ± 0.23	1.60 ± 1.17
Met-*p*NA	2.55 ± 0.34	1.27 ± 0.88
Val-*p*NA	0.54 ± 0.14	1.52 ± 0.01
Ala-*p*NA	0.89 ± 0.68	2.79 ± 0.25
Leu-*p*NA	1.83 ± 0.22	1.35 ± 0.39
Pro-*p*NA	0.78 ± 0.28	3.04 ± 0.24
Gly-*p*NA	0.12 ± 0.10	1.60 ± 0.14
Thr-*p*NA	0.27 ± 0.07	5.92 ± 0.41

^A^ Mn^2+-^activated DAPs were incubated amino acyl-*p*NA substrates (5 mM) at 37°C for 10 min. Hydrolysis of substrates was determined as described in *Experimental Procedures*. Relative activities with standard deviations are expressed as % of the Asp-*p*NA reaction. Reactions were performed in triplicate and the mean from three experiments is shown.

**Fig 6:**
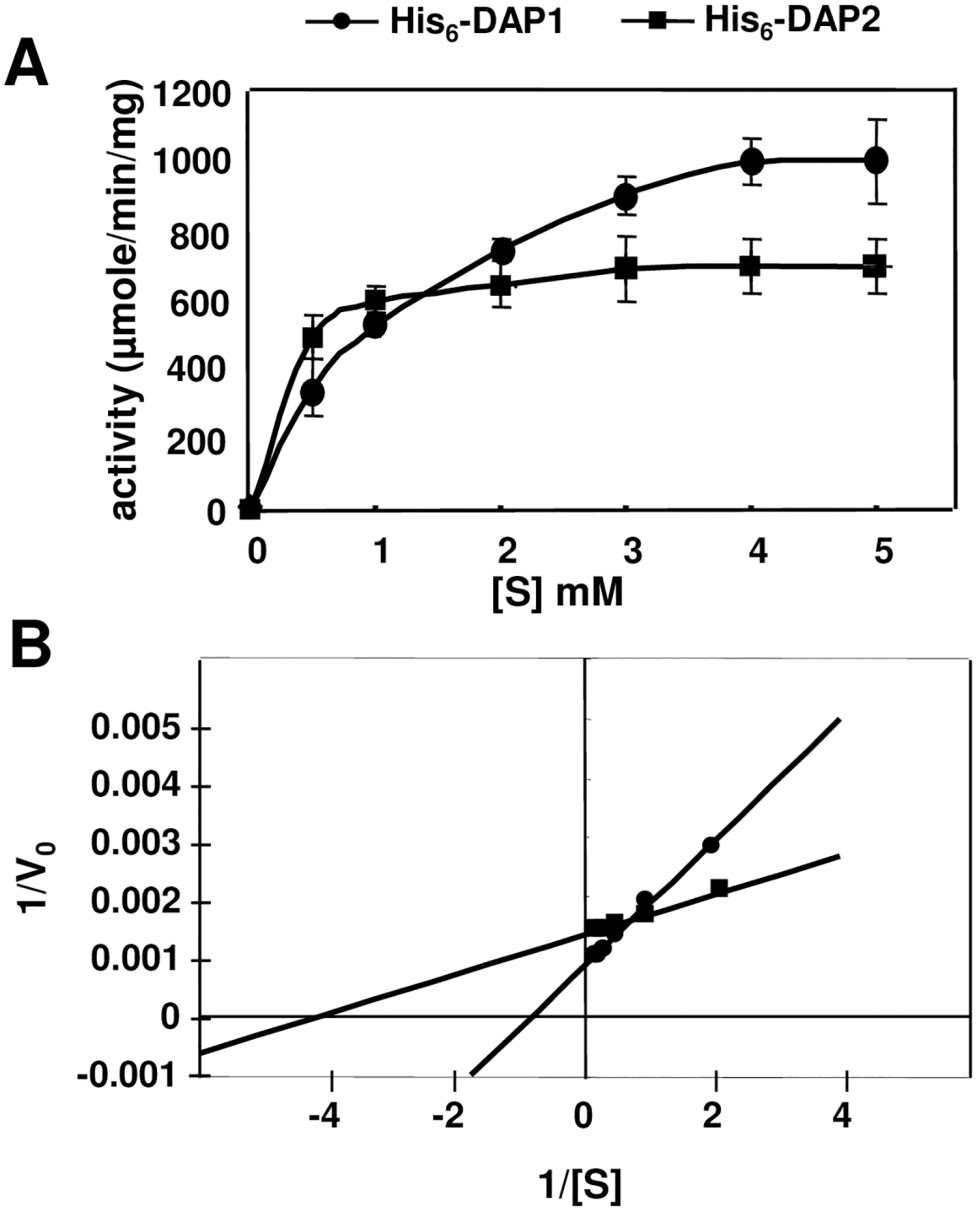
AtDAP1 and AtDAP2 kinetics. *A*, Initial rates of substrate hydrolysis by His_6_-DAP1 (●) and His_6_-DAP2 (■) were determined using 0.5–5 mM Asp-*p*NA at pH 8.0. *B*, *K*_*m*_ and *V*_*max*_ for His_6_-DAP1 (●) and His_6_-DAP2 (■) were determined from the Lineweaver-Burk plots.

Mn^2+^-activated His_6_-DAP1 and His_6_-DAP2 and Asp-*p*-NA (0.25 to 5 mM) were used to determine initial velocities of hydrolysis (Fig 6A). Lineweaver-Burk plots were used to determine the *K*_m_ and *V*_max_ of His_6_-DAP1 and His_6_-DAP2 (Fig 6B) revealing differences in the two *Arabidopsis* DAPs. His_6_-DAP2’s *V*_max_ (714.8 μmol min^-1^ mg^-1^) and *k*_cat_ (7283 sec^-1^) values were 1.7-fold lower than His_6_-DAP1’s *V*_max_ and *k*_cat_ (Table 4). However, since His_6_-DAP2’s *K*_m_ (0.21 mM) was six-fold lower than His_6_-DAP1 (1.25 mM), this translated into a 3.3-fold higher catalytic efficiency (k_cat_/*K*_m)_ for His_6_-DAP2 (3.47 x 10^7^ M^-1^ sec^-1^) relative to His_6_-DAP1 (1.0 x 10^7^ M^-1^ sec^-1^) (Table 4).

**Table 4:** Kinetic parameters for hydrolysis of Asp-pNA by His_6_-DAP1 and His_6_-DAP2^A^.

Enzyme	Vmax (mmol min^-1^ mg^-1)^	Km (mM)	kcat (sec^-1^)	Kcat/Km (sec^-1^ M^-1^)
AtDAP1	1250	1.25	13106	10.5 x 10^6^
AtDAP2	714.8	0.21	7283	34.7 x 10^6^

### AtDAP1 is a molecular chaperone

**Fig 7:**
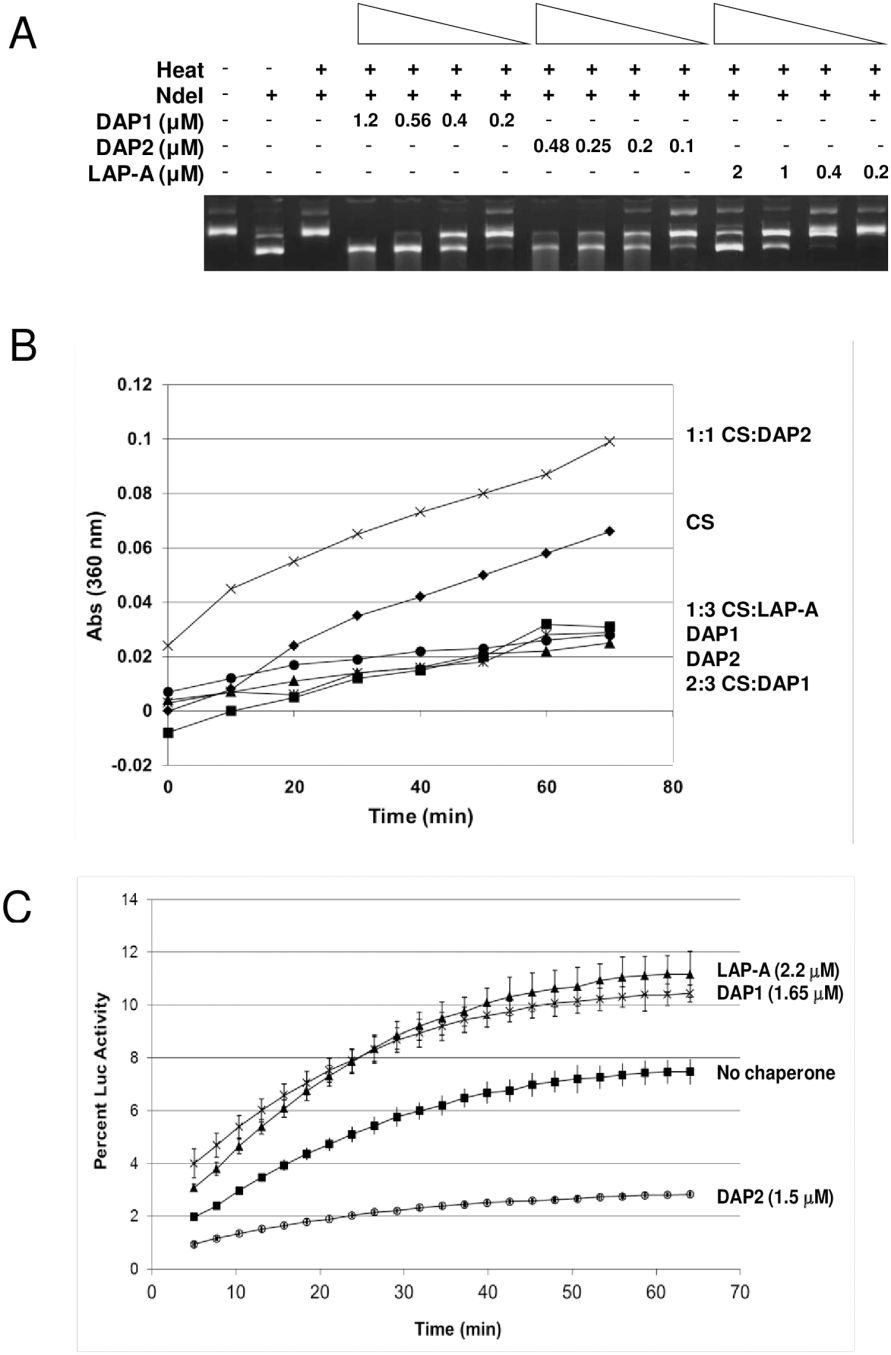
AtDAP1 and AtDAP2 chaperone activity. A, Thermal protection assay. NdeI (1 U) was incubated in the presence or absence of His_6_-DAP1 (0.2–1.2 μM), His_6_-DAP2 (0.1–48 μM; *A*) or His_6_-LAP-A (0.2–2 μM) for 90 min at 43°C. At this time, 140 ng of plasmid DNA was added and digested for 90 min at 37°C. Control lanes show plasmid DNA only and DNA after digestion with unheated NdeI. NdeI cuts at two sites releasing fragments 4.6 kb and 0.2 kb; only the 4.6-kb fragment is shown on these gels. The monomeric supercoiled plasmid (SC) and multimeric supercoils are observed in undigested DNA samples. *B*, Thermal aggregation assay. CS (300 nM) was incubated with His_6_-DAP1 (600 nM; ▲), His_6_-DAP2 (300 nM; x), or His_6_-LAP-A (900 nM; ■) or without chaperone (♦) at 43°C for 60 min. These concentrations corresponded to CS: DAP1, CS: DAP2 and CS:LAP-A ratios of 2:3, 1:1, and 1:3, respectively. Neither His_6_-DAP1 (✳) nor His_6_-DAP2 (●) aggregated on their own after heating. Aggregation of CS was determined by measuring light scattering at 360 nm. *C*, Luc refolding assay. Luc (1 μM) was heated for 11 min at 42°C with 1.5 μM His_6_-DAP1 (x), 1.5 μM His_6_-DAP2 (○), or 2.2 μM His_6_-LAP-A (▲), or no chaperone (■). Luc was allowed to refold in the presence of rabbit reticulocyte lysate (RRL) supplemented with 2 mM ATP. Percent activity corresponds to the relative luminescence compared to unheated luciferase. Measurements were taken for three technical replicates. Data for all panels are representative of two or more independent experiments.

His_6_-DAP1 and His_6_-DAP2 were tested for their ability act as molecular chaperones using three established assays as described previously [35]. The ability of the His_6_-DAPs to protect NdeI from thermal inactivation was assessed (Fig 7A). Both His_6_-DAP1 and His_6_-DAP2 protected NdeI at concentrations as low as 0.2 μM, suggesting both proteins are more potent molecular chaperones than the bifunctional leucine aminopeptidases of tomato and *Arabidopsis* and small heat shock proteins [35] and its chaperone activity was similar to the SpDAP [56].

The ability of His_6_-DAP1 and His_6_-DAP2 to prevent thermal-induced aggregation of citrate synthase (CS) differed (Fig 7B). Only His_6_-DAP1 (600 nM) exhibited clear molecular chaperone activity, reducing CS aggregation by ~40–60%. Similar activities were seen with plant LAPs and a yeast DAP [35, 56]. In contrast, His_6_-DAP2 (300 nM) actually increased CS aggregation, indicating that His_6_-DAP2 did not display chaperone activity in this assay.

Finally, the luciferase (Luc) activity assay assessed the ability of DAPs to promote Luc refolding (Fig 7C). Luc was heated to 43°C in the presence or absence of His_6_-DAP1 or His_6_-DAP2 and then allowed to refold aided by the ATP-dependent chaperones in rabbit reticulocyte lysates. His_6_-DAP1 promoted ~10% of the Luc to refold, which was similar to the positive control LAP-A [35]. Surprisingly, His_6_-DAP2 inhibited Luc refolding.

## Discussion

### Green plant DAP gene families have expanded, contracted and diversified

Animal, fungal, apicomplexan, bacterial, and oomycete genomes harbor single *DAP* genes. In contrast, during the evolution of green plants (green algae and land plants), there is evidence for ancient and more recent *DAP* gene duplications, as well as *DAP* gene losses. The first *DAP* gene duplication likely occurred in an ancestral, flagellated green algae approximately 1500 Mya [46, 47]. This is supported by the fact that single *DAP* genes were found in both red and glaucophyte algae, while two classes of *DAPs* (*DAP1* and *DAP2*) were found in land plants.

As the Chlorophyte algae and land plants diverged from their ancestral green algae, different and independent *DAP* gene evolutionary events occurred. Land plants retained *DAP1* genes and there is evidence for recent independent *DAP1* gene duplications in the clubmoss (*S*. *moellendorffii*) and poplar (*P*. *trichocarpa*). In contrast, the *DAP1* lineage was lost in Trebouxiophyceae (*C*. *variabilis* and *C*. *subellipsoidea*) and Chlorophyceae algae (*C*. *reinhardtii*), as well as the green picoeukaryotic algae. These data indicate that a *DAP1* gene was not essential for life as single-celled green algae.

Early in chlorophyte evolution, there was a duplication of an ancestral *DAP* gene that gave rise to the *DAP2* lineage. This is supported by green picoeukaryotic algae genomes harboring both a *DAP2* gene and a more primitive *DAP2-*like gene. This *DAP2* gene was subsequently lost in green algae, while the ancestral *DAP2*-like gene was retained. In contrast, vascular and non-vascular land plants have lost the primitive *DAP2*-like gene. Land plants have one or more *DAP2* genes. Recent independent *DAP2* gene duplications were noted in the clubmoss (*S*. *moellendorffii)* and moss (*P*. *patens*). These data indicate that multiple *DAP2/DAP2-like* genes are not essential for “being green”.

### Plant DAPs are located in the chloroplast and vacuole

Based on predicted N-terminal transit peptides and proteomics [52], the eudicot DAP2 proteins are predicted to be plastid localized (S3 Table) and their chloroplast stroma localization is confirmed by Ferro et al [52]. In contrast, these programs (TargetP, Predator, Plant-mPLoc) predict a mitochondrial or dual chloroplast/ mitochondrial location for the DAP2s from maize, rice, barley, and *Brachypodium*. Further investigations into the DAP2 subcellular localizations are needed to: (1) resolve whether current algorithms accurately predict monocot protein localizations in mitochondria and chloroplasts; (2) determine if monocot and dicot DAP2s reside in the same or different subcellular compartments; and (3) determine if monocot DAP2s will join the ranks of dual-localized proteins [57, 58].

Plant DAP1 proteins do not contain an N-terminal peptide directing them to a subcellular location making them more similar to the yeast DAP (Ape4, Yhr113w) and animal DNPEPs (S3 Table). Two proteomics studies have confidently reported AtDAP1 in vacuolar preparations [53, 54]. In addition, there is substantial proteomic, biochemical and cellular evidence that the yeast DAP (Ape4) is located in both the cytosol and vacuole [59, 60]. During nutrient deprivation, Ape4 is a Cyt (cytoplasmic to vacuole) cargo and is dependent on Atg19 for transport to the vacuole [60]. While many components of the Cyt macroautophagy system of yeast, animals and plants are conserved [61], it is noteworthy that higher plants do not have Atg19-like proteins, nor well-studied Atg19 cargos Ape1 (Aminopeptidase 1) or Ams1 (α-mannosidase) (data not shown). If a selective autophagy mechanism is active in the transport of DAP1 to vacuoles, it is likely to be distinct for higher plants. *DAP1* proteins and RNAs are ubiquitous and found in numerous organs [62–66]. In fact, based on AtGenExpress datasets [63–65] and the expression browsers at the Bio-Analytic Resource [66], the *AtDAP1* and *AtDAP2* RNAs are unlikely to be modulated during development, biotic stress or abiotic stress, including nutrient deprivation.

### DAPs have biochemically diverged and conserved features

Although the subcellular locations of the plant DAP1 and DAP2 are distinct from their eukaryotic DAP counterparts, the proteins are remarkably conserved when key residues critical for enzymatic function and assembly are examined. Like other DAPs, the AtDAP1 and AtDAP2 are dodecamers. Virtually all residues that have been implicated in divalent cation coordination, substrate binding, flexible loop inter-digitation with adjacent subunits, and catalysis that were identified by X-ray crystal structures or mutagenesis are conserved in plant DAP1 and DAP2 proteins.

Similar to the microbial and animal DAPs, AtDAP1 and AtDAP2 are metallopeptidases as they are both strongly inhibited by EDTA and moderately inhibited by 1,10-phenanthroline. Similar responses to both EDTA and 1,10-phenanthroline were displayed by the DAPs from fungi (yeast and *A*. *oryzea*), *Plasmodium* and mouse [10, 16, 18, 67]. In contrast, other DAPs were preferentially inhibited by one chelator but not the other [12, 55]. These different responses to chelators may reflect the redox state of residues within the catalytic site. As expected for metallopeptidases, addition of divalent cations stimulated AtDAP1 and AtDAP2 activity, with both enzymes maximally active in the presence of Mn^2+^. While, Mg^2+^ and Ca^2+^ stimulated AtDAP2 activity, these ions had no impact on AtDAP1. Finally, like other DAPs, Zn^2+^ strongly inhibited DAP1 and DAP2 activities [5, 12, 13, 17, 18, 55, 67]; the ability of exogenous zinc to inhibit metallopeptidases, as well as other peptidases, is well documented [68].

Two peptidase inhibitors (anti-thrombin III and PMSF) inhibited DAP1 or DAP2 activities. Anti-thrombin III is a Ser protease inhibitor, while PMSF is an inhibitor of Ser and some Cys proteases. In their catalytic domains, there are two conserved Ser residues; one is implicated in cation binding (i.e., AtDAP1 Ser86) and the other (i.e., AtDAP1 Ser416) is located within the catalytic pocket with a unknown role in DAP’s proteolytic mechanism. It is also noteworthy that the C-terminal proteolytic domain is Ser rich and harbors the one known phosphorylated Ser residue (i.e., AtDAP1’s Ser284) [43, 44]. At the present time, which (if any) of the Ser residues are critical for the activity of *Arabidopsis* DAPs is not known. Surprisingly, few studies have tested the impact of Ser protease inhibitors on DAPs and it is not clear if a Ser residue plays an important role only in plant DAPs or in DAPs from other kingdoms.

The thiol-reducing agent DTT stimulated Mn^2+^-activated DAP1 and DAP2 by 2.4 fold. The response of the plant DAPs to DTT is distinct from responses of the mammalian DNPEPs, which were either inhibited by DTT [10, 12] or unaffected [14]. DTT does not impact the human DNPEP’s quaternary structure [12], implying that thiols or redox state may have a role in catalysis. Overall DAPs have a relatively small number of Cys residues (between 8 and 11) but there are two highly conserved Cys residues (eg., AtDAP1’s Cys271 and Cys414) in the C-terminal proteolytic domain. As a number of thiol-activated metallopeptidases and Ser peptidases have been previously described [68], it will be of value to test the role of these residues in catalysis in the future.

There are several additional features that distinguish the plant DAP1 and DAP2 proteins. First, the pH optimum of AtDAP1 is not well aligned with its subcellular localization (cytosol or vacuole) [53, 54]. AtDAP1’s pH optima is 8.5, while the pH of the vacuole is 6.3 [69]. It is possible that AtDAP1 is active after cellular damage when cellular contents are mixed and the pH rises closer to the AtDAP1 optima. In contrast, the chloroplast-localized AtDAP2 has a pH optimum of 8.0, which is close to the pH of the plastid stroma, which ranges from 7.1 to 8.0 depending on photosynthetic activities. For comparisons, most mammalian and the *Plasmodium* DAPs have pH optima as 7.5 [9, 10, 12, 70], while fungal DAPs had slightly higher pH optima ranging from 7.5 to 9.0 [16, 55, 67].

Second, of the DAPs characterized to date, AtDAP1 is most temperature resistant with its peak activity at 60°C. AtDAP2, the *Plasmodium* M18AAP and *Aspergillus* DAP have temperature optima of 40°C, 33–39°C, and 50°C, respectively [70]. The significance of a higher temperature range is not clear, as *Arabidopsis DAP1* RNAs are not induced in response to heat stress based on microarray data at BAR and AtGenExpress [64, 66].

Third, both AtDAP1 and AtDAP2 prefer substrates with N-terminal Asp over N-terminal Glu. Like the plant DAPs, the mammalian DAPs from readily hydrolyze substrates with acidic residues at the N-terminus, with a preference for Asp over Glu residues [9, 10, 55]. However, the *Plasmodium* M18AAP, yeast Ape4, and *Aspergillus* DAP expressed in *E*. *coli*, prefer Glu over Asp and the *P*. *aeruginosa* M18AAP hydrolyzes both acidic substrates at equivalent rates [5, 16, 18, 67]. The *Arabidopsis* DAPs ready hydrolyze chromogenic substrates, whereas, the literature suggests that M18 DAPs differ in their ability to cleave chromogenic/fluorometric substrates and peptides in vitro. Some DAPs prefer peptide substrates [10, 12, 13, 16], while others will readily hydrolyze chromogenic or fluorometric substrates, as well as peptides [5, 14, 18, 55, 67]. For example, the mouse brain DAP cleaves chromogenic substrates 200-fold less efficiently compare to Asp-Ala substrate [10].

Finally, using their optimal chromogenic substrate (Asp-*p*-NA), the Mn^2+-^activated DAP1 and DAP2 displayed different *K*_m_, *V*_max_ and *k*_cat_ values. Both AtDAP1 and AtDAP2 had high catalytic efficiencies, 1.05 x 10^7^ M^-1^ sec^-1^ and 3.47 x10^7^ M^-1^ sec^-1^, respectively. There are only two reports of DAP kinetic parameters on non-peptide substrates [5, 70]. The *P*. *aeruginosa* DAP, was also assayed on Asp-*p*-NA in the presence of Mn^2+^ and its *k*_cat_/*K*_m_ was 7800 M^-1^ sec^-1^; while the *Plasmodium* M18AAP had a catalytic efficiency of 1081 M^-1^ sec^-1^ when its substrate was Asp-NHMec (Asp-7-amido-4-methyl-coumarin). The highest *k*_cat_/*K*_m_ values that have been reported for mammalian DNPEPs were revealed using peptide and peptide hormones as substrates [12–14]. For example, using Angiotensin II (Asp-Arg-Val-Tyr-Ile-His-Pro-Phe) as a substrate, *k*_cat_/*K*_m_ values of 2.7 x 10^4^ M^-1^ sec^-1^ and 7.6 x 10^4^ M^-1^ sec^-1^ were reported for the native rabbit DNPEP and Mn^2+^-activated bovine His_6_-DNPEP, respectively [12, 14].

### Substrates—What do DAPs do in vivo?

Mammalian DNPEPs have received substantial attention in recent years due to their ability to hydrolyze angiotensinogen-derived peptides from the renin-angiotensin system, which controls blood pressure homeostasis [7, 12, 14]. Potential regulatory roles for DNPEPs in neurons and the bone morphogenetic protein (BMP)-signaling pathway have also been recently suggested [14, 19, 71]. There is also substantial evidence that mammalian DAPs have an important regulatory role in endocytic vesicle sorting and recycling [11] and are implicated in albumin uptake in renal proximal tubules [72]. Finally, the yeast DAP (Ape4) subcellular localization responds to nutritional status, as more Ape4 is vacuolar localized during times of nutrient stress. Therefore, like other Atg19 cargos, Ape4 may contribute to turnover of proteins and peptides within the vacuole during times of nutrient limitation [60]. The *Plasmodium* M18AAP also has a role in nutrition [18]; in M18AAP-knock down mutants, *Plasmodium* have ruptured food vacuoles, suggesting that *DAP*-deficient *Plasmodium* are unable to digest food vacuole-localized proteins and peptides.

The *Arabidopsis* DAP1 is located with the plant vacuole [53, 54]. If AtDAP1 has a role in controlling vesicle or vacuolar cargos, the role is not essential as *dap1* mutants are viable and did not display obvious phenotypes during growth and development (unpublished results). Similarly, the plastid-localized DAP2 is not essential. Given their distinct subcellular locations, DAP1’s and DAP2’s substrates are likely distinct and remain to be discovered.

In considering DAP1’s mechanism of action, we must consider the fact AtDAP1 is bifunctional. Like the *S*. *pombe* DAP [56] and LAPs of tomato and *Arabidopsis* [35], AtDAP1 is a both an aminopeptidase and a potent molecular chaperone. By virtue of its aminopeptidase activity and localization in vacuoles, AtDAP1 may be critical in the biogenesis of plant bioactive peptides or involved in protein and peptide turnover in the cytosol and/or vacuole [73, 74]. It is intriguing to speculate that DAP1’s ability to remove acidic residues from peptide/protein substrates might influence peptide stability based on Asp and Glu being primary destabilizing residues based on the N-end rule [75]. Alternatively, its molecular chaperone activity may refold target proteins in the cytosol and/or vacuole prolonging their activities prior to turnover.

While AtDAP2 is an aminopeptidase, its status as a molecular chaperone is less certain. AtDAP2 only displayed chaperone activity in the NdeI thermal protection assay. The inability of AtDAP2 to display chaperone-like activities in the CS aggregation assay and Luc refolding assays could be attributed to the relative temperature sensitivity of His_6_-DAP2 (Fig 5B). All three molecular chaperone assays incubated substrates at 43°C for different times for substrate inactivation (NdeI 90 min, CS 60 min, Luc 11 min) and used different temperatures for the assays (NdeI 37°C, CS 43°C, Luc 30°C). As there was no correlation with incubation periods at 43°C or the temperature of the chaperone assays, it is not clear why AtDAP2 displayed molecular chaperone activity in the NdeI assay but not the CS and Luc assays. It is possible that the 90-min incubation at 37°C conditions that were used in the thermal protection assay allowed AtDAP2 to resume its native structure and perform its molecular chaperone function.

Determining the identity of the *in vivo* peptidase substrates and chaperone clients of AtDAP1 and AtDAP2 should be a priority in the future. These studies will reveal if AtDAP1 peptidase and chaperone clients will be one and the same. It is attractive to hypothesize that chaperone activity may be an inherent property of multimeric aminopeptidases. The bifunctional DAPs and LAPs do not have the conserved α-crystallin domains of heat-shock proteins [76], nor do they share significant identity with each other. Therefore, it is likely that novel chaperone domains must be active in these bifunctional proteins. As postulated for plant LAPs, the chaperone domain may enable docking of substrates for hydrolysis or alternatively enable the assembly of subunits into their multimeric structures [35, 77].

Recent crystal structures of the tomato wild-type LAP-A, which is peptidase and chaperone active, and a catalytic mutant K354A, which is peptidase inactive and chaperone enhanced, was determined [35, 77–79]. The LAP-A K354A hexamer spontaneously disassembles into trimers, dimers and monomers. The K354A mutation causes conformation changes in the catalytic pocket and also creates a small mobile loop at the interface of LAP-A trimers. This loop prevents the K354A subunits from stable hexamer assembly and exposes hydrophobic surfaces [77]. It is postulated that these hydrophobic surfaces may be transiently exposed as wild-type LAP-A subunits form trimers, which are then stacked into the active LAP-A hexamer. These surfaces may contribute to LAP-A’s chaperone function in vivo and in vitro. Therefore, it is possible that the interfaces of the DAP1 dimers, which assemble into the dodecamer, may also contribute to DAP1’s chaperone activity. Mutational analysis of DAP1 will identify the residues associated with chaperone activity and determine if like tomato’s LAP-A, chaperone activity is independent of its peptidase activity.

## Supporting information

S1 Table Roles of conserved residues in the Arabidopsis, human, bovine, *P*. *aeruginosa*, and *Plasmodium* DAPs. (DOCX)

S2 Table Musite-predicted Ser and Thr phosphorylation sites in plant DAP1 and DAP2 proteins. (DOCX)

S3 Table Chlorophyte DAP genes and DAPs from other selected species. (PDF)

## Abbreviations

2D-PAGE: 2-dimensional-polyacrylamide gel electrophoresis
BSA: bovine serum albumin
CS: citrate synthase
DAP: aspartyl aminopeptidase
DMSO: dimethylsulfoxide
DTT: dithiothreitol
GUS: β-glucuronidase
IPTG: isopropyl β-D-1-thiogalactopyranoside
Luc: luciferase
NTA: nitrilotriacetic acid
*p*-NA: *p*-nitroanilide
RRL: rabbit reticulocyte lysate
X-Glu: 5-bromo-4-chloro-3-indolyl-β-D-glucopyranoside

## References

[1] Broder DH, Miller CG. DapE can function as an aspartyl peptidase in the presence of Mn^2+^. J Bact. 2003;185:4748–4754. doi: 10.1128/JB.185.16.4748-4754.2003 12896993 10.1128/JB.185.16.4748-4754.2003 PMC166485

[2] Carter TH, Miller CG. Asparate-specific peptidases in *Salmonella typhimurium* mutants deficient in peptidase E. J Bact. 1984;159:453–459. 6086568 10.1128/jb.159.2.453-459.1984 PMC215666

[3] Larsen RA, Knox TM, Miller CG. Aspartic peptide hydrolases in *Salmonella enterica* serovar typhimurium. J Bact. 2001;183:3089–3097. doi: 10.1128/JB.183.10.3089-3097.2001 11325937 10.1128/JB.183.10.3089-3097.2001 PMC95209

[4] Mathew Z, Knox TM, Miller CG. *Salmonella enterica* serovar typhimurium peptidase B is a leucyl aminopeptidase with specificity for acidic amino acids. J Bact. 2000;182:3383–3393. 10852868 10.1128/jb.182.12.3383-3393.2000 PMC101900

[5] Nguyen DD, Pandian R, Kim D, Ha SC, Yoon H-J, Kim KS, et al Structural and kinetic bases for the metal preference of the M18 aminopeptidase from *Pseudomonas aeruginosa*. Biochem Biophys Res Comm. 2014;447:101–107. doi: 10.1016/j.bbrc.2014.03.109 24704201 10.1016/j.bbrc.2014.03.109

[6] Banegas I, Prieto I, Vives F, Alba F, de Gasparo M, Segarra AB, et al Brain aminopeptidases and hypertension. J Renin-Angio-Aldos Sys. 2006;7:129–134. 10.3317/jraas.2006.021 17094048

[7] Wright JW, Harding JW. Brain renin-angiotensin—A new look at an old system. Prog Neurobiol. 2011;95:49–67. doi: 10.1016/j.pneurobio.2011.07.001 21777652 10.1016/j.pneurobio.2011.07.001

[8] Glenner GG, McMillan PJ. A mammalian peptidase specific for the hydrolysis of N-terminal α-L-glutamyl and aspartyl residues. Nature. 1962;194:867. 10.1038/194867a0 13899213

[9] Cheung HS, Cushman DW. A soluble aspartate aminopeptidase from dog kidney. Biochim Biophys Acta. 1971;242:190–193. 4330623 10.1016/0005-2744(71)90098-2

[10] Kelly JA, Neidle EL, Neidle A. An aminopeptidase from mouse brain cytosol that cleaves N-terminal acidic amino acid residues. J Neurochem. 1983;40:1727–1734. 6854330 10.1111/j.1471-4159.1983.tb08148.x

[11] Li X, Chen BH, Yoshina S, Cai TX, Yang FQ, Mitani SH, et al Inactivation of *Caenorhabditis elegans* aminopeptidase DNPP-1 restores endocytic sorting and recycling in *tat-1* mutants. Mol Biol Cell. 2013;24:1163–1175. doi: 10.1091/mbc.E12-10-0730 23427264 10.1091/mbc.E12-10-0730 PMC3623637

[12] Wilk S, Wilk E, Magnusson RP. Purification, characterization, and cloning of a cytosolic aspartyl aminopeptidase. J Biol Chem. 1998;273:15961–15970. 9632644 10.1074/jbc.273.26.15961

[13] Wilk S, Wilk E, Magnusson RP. Identification of histidine residues important in the catalysis and structure of aspartyl aminopeptidase. Arch Biochem Biophys. 2002;407:176–183. 12413488 10.1016/s0003-9861(02)00494-0

[14] Chen YY, Farquhar ER, Chance MR, Palczewski K, Kiser PD. Insights into substrate specificity and metal activation of mammalian tetrahedral aspartyl aminopeptidase. J Biol Chem. 2012;287:13356–13370. doi: 10.1074/jbc.M112.347518 22356908 10.1074/jbc.M112.347518 PMC3339940

[15] Iribar C, Esteban MJ, Martinez JM, Peinado JM. Decrease in cytosolic aspartyl-aminopeptidase but not in alanyl-aminopeptidase activity in the frontal-cortex of the aged rat. Brain Res. 1995;687:211–213. 7583308 10.1016/0006-8993(95)00538-2

[16] Yokoyama R, Kawasaki H, Hirano H. Identification of yeast aspartyl aminopeptidase gene by purifying and characterizing its product from yeast cells. FEBS J. 2006;273:192–198. doi: 10.1111/j.1742-4658.2005.05057.x 16367759 10.1111/j.1742-4658.2005.05057.x

[17] Sivaraman KK, Oellig CA, Huynh K, Atkinson SC, Poreba M, Perugini MA, et al X-ray crystal structure and specificity of the *Plasmodium falciparum* malaria aminopeptidase PfM18AAP. J Mol Biol. 2012;422:495–507. doi: 10.1016/j.jmb.2012.06.006 22709581 10.1016/j.jmb.2012.06.006

[18] Teuscher F, Lowther J, Skinner-Adams TS, Spielmann T, Dixon MWA, Stack CM, et al The M18 aspartyl aminopeptidase of the human malaria parasite *Plasmodium falciparum*. J Biol Chem. 2007;282:30817–30826. doi: 10.1074/jbc.M704938200 17720817 10.1074/jbc.M704938200

[19] Chaikuad A, Pilka ES, De Riso A, von Delft F, Kavanagh KL, Venien-Bryan C, et al Structure of human aspartyl aminopeptidase complexed with substrate analogue: insight into catalytic mechanism, substrate specificity and M18 peptidase family. BMC Struc Biol. 2012;12:14 10.1186/1472-6807-12-14 PMC3472314 22720794

[20] Gabriel M, Telmer PG, Marsolais F. Role of asparaginase variable loop at the carboxyl terminal of the alpha subunit in the determination of substrate preference in plants. Planta. 2012;235:1013–1022. doi: 10.1007/s00425-011-1557-y 22127737 10.1007/s00425-011-1557-y

[21] Hejazi M, Piotukh K, Mattow J, Deutzmann R, Volkmer-Engert R, Lockau W. Isoaspartyl dipeptidase activity of plant-type asparaginases. Biochem J. 2002;364:129–136. 11988085 10.1042/bj3640129 PMC1222554

[22] Asano M, Nakamura N, Kawai M, Miwa T, Nio N. Purification and characterization of an N-terminal acidic amino acid-specific aminopeptidase from soybean cotyledons (*Glycine* max). Biosci Biotech Biochem. 2010;74:113–118. 10.1271/bbb.90617 20057138

[23] Walling LL. Recycling or regulation? The role of amino-terminal modifying enzymes. Curr Opin Plant Biol. 2006;9:227–233. doi: 10.1016/j.pbi.2006.03.009 16597508 10.1016/j.pbi.2006.03.009

[24] Emanuelsson O, Brunak S, von Heijne G, Nielsen H. Locating proteins in the cell using TargetP, SignalP and related tools. Nature Proto. 2007;2:953–971. 10.1038/nprot.2007.131 17446895

[25] Emanuelsson O, Nielsen H, Von Heijne G. ChloroP, a neural network-based method for predicting chloroplast transit peptides and their cleavage sites. Prot Sci. 1999;8:978–984. 10.1110/ps.8.5.978 PMC2144330 10338008

[26] Small I, Peeters N, Legeai F, Lurin C. Predotar: A tool for rapidly screening proteomes for N-terminal targeting sequences. Proteomics. 2004;4:1581–1590. doi: 10.1002/pmic.200300776 15174128 10.1002/pmic.200300776

[27] Chou K-C, Shen H-B. Plant-mPLoc: A top-down strategy to augment the power for predicting plant protein subcellular localization PLoS ONE. 2010;5:e11335 doi: 10.1371/journal.pone.0011335 20596258 10.1371/journal.pone.0011335 PMC2893129

[28] Walker NS, Stiffler N, Barkan A. POGs/PlantRBP: a resource for comparative genomics in plants. Nuc Acids Res. 2007;35:D852–D856. 10.1093/nar/gkl795 PMC1669711 17142226

[29] Notredame C, Higgins DG, Heringa J. T-Coffee: A novel method for fast and accurate multiple sequence alignment. J Mol Biol. 2000;302:205–217. doi: 10.1006/jmbi.2000.4042 10964570 10.1006/jmbi.2000.4042

[30] Do CB, Mahabhashyam MS, Brudno M, Batzoglou S. ProbCons: Probabilistic consistency-based multiple sequence alignment. Genome Res. 2005;15:330–340. doi: 10.1101/gr.2821705 15687296 10.1101/gr.2821705 PMC546535

[31] Capella-Gutierrez S, Silla-Martinez JM, Gabaldon T. trimAl: a tool for automated alignment trimming in large-scale phylogenetic analyses. Bioinformatics. 2009;25:1972–1973. doi: 10.1093/bioinformatics/btp348 19505945 10.1093/bioinformatics/btp348 PMC2712344

[32] Nguyen LT, Schmidt HA, von Haeseler A, Minh BQ. IQ-TREE: a fast and effective stochastic algorithm for estimating maximum-likelihood phylogenies. Mol Biol Evol. 2015;32:268–274. doi: 10.1093/molbev/msu300 25371430 10.1093/molbev/msu300 PMC4271533

[33] Shimodaira H, Hasegawa M. Multiple comparisons of log-likelihoods with applications to phylogenetic inference. Mol Biol Evol. 1999;16:1114.

[34] Pautot V, Holzer FM, Walling LL. Differential expression of tomato *Proteinase Inhibitor I* and *Inhibitor II* genes during bacterial pathogen invasion and wounding. Mol Plant-Microbe Inter. 1991;4:284–292. 10.1094/mpmi-4-284 1932815

[35] Scranton M, Yee A, Park SY, Walling LL. Plant leucine aminopeptidases moonlight as molecular chaperones to alleviate stress-induced protein damage. J Biol Chem. 2012;287:18408–18417. doi: 10.1074/jbc.M111.309500 22493451 10.1074/jbc.M111.309500 PMC3365729

[36] Gu YQ, Holzer FM, Walling LL. Overexpression, purification and biochemical characterization of the wound-induced leucine aminopeptidase of tomato. Eur J Biochem. 1999;263:726–735. 10469136 10.1046/j.1432-1327.1999.00548.x

[37] Hedrick JL, Smith AJ. Size and charge isomer separation and estimation of molecular weights of proteins by disc gel electrophoresis. Arch Biochem Biophys. 1968;126:155–164. 5671059 10.1016/0003-9861(68)90569-9

[38] Bryan JK. Molecular weights of protein multimers from polyacrylamide gel electrophoresis. Anal Biochem. 1977;78:513–519. 851223 10.1016/0003-2697(77)90111-7

[39] Ellis KJ, Morrison JF. Buffers of constant ionic strength for studying pH-dependent processes. Meth Enzymol. 1982;87:405–426. 7176924 10.1016/s0076-6879(82)87025-0

[40] Marchler-Bauer A, Anderson JB, Chitsaz F, Derbyshire MK, DeWeese-Scott C, Fong JH, et al CDD: specific functional annotation with the Conserved Domain Database. Nucl Acids Res. 2009;37:D205–210. doi: 10.1093/nar/gkn845 18984618 10.1093/nar/gkn845 PMC2686570

[41] Metz G, Rohm KH. Yeast aminopeptidase. 1. Chemical composition and catalytic properties. Biochim Biophys Acta. 1976;429:933–949. 5147 10.1016/0005-2744(76)90338-7

[42] Yao Q, Gao J, Bollinger C, Thelen J, Xu D. Predicting and analyzing protein phosphorylation sites in plants using Musite. Front Plant Sci. 2012;3:186 doi: 10.3389/fpls.2012.00186 22934099 10.3389/fpls.2012.00186 PMC3423629

[43] Durek P, Schmidt R, Heazlewood JL, Jones A, Maclean D, Nagel A, et al PhosPhAt: the *Arabidopsis thaliana* phosphorylation site database. An update. Nuc Acids Res. 2010;38:D828–D834. 10.1093/nar/gkp810 PMC2808987 19880383

[44] Sugiyama N, Nakagami H, Mochida K, Daudi A, Tomita M, Shirasu K, et al Large-scale phosphorylation mapping reveals the extent of tyrosine phosphorylation in Arabidopsis. Mol Sys Biol. 2008;4:193 10.1038/msb.2008.32 PMC2424297 18463617

[45] Reyes-Prieto A, Bhattacharya D. Phylogeny of nuclear-encoded plastid-targeted proteins supports an early divergence of glaucophytes within plantae. Mol Biol Evol. 2007;24:2358–2361. doi: 10.1093/molbev/msm186 17827169 10.1093/molbev/msm186

[46] Lewis LA, McCourt RM. Green algae and the origin of land plants. Amer J Bot. 2004;91:1535–1556. 21652308 10.3732/ajb.91.10.1535

[47] Leliaert F, Smith DR, Moreau H, Herron MD, Verbruggen H, Delwiche CF, et al Phylogeny and molecular evolution of the green algae. Crit Rev Plant Sci. 2012;31:1–46.

[48] Becker B. Snow ball earth and the split of Streptophyta and Chlorophyta. Trends Plant Sci. 2013;18:180–183. doi: 10.1016/j.tplants.2012.09.010 23102566 10.1016/j.tplants.2012.09.010

[49] Becker B, Marin B. Streptophyte algae and the origin of embryophytes. Ann Bot. 2009;103:999–1004. doi: 10.1093/aob/mcp044 19273476 10.1093/aob/mcp044 PMC2707909

[50] Proost S, Pattyn P, Gerats T, Van de Peer Y. Journey through the past: 150 million years of plant genome evolution. Plant J. 2011;66:58–65. doi: 10.1111/j.1365-313X.2011.04521.x 21443623 10.1111/j.1365-313X.2011.04521.x

[51] Gill N, Findley S, Walling JG, Hans C, Ma J, Doyle J, et al Molecular and chromosomal evidence for allopolyploidy in soybean. Plant Physiol. 2009;151:1167–1174. doi: 10.1104/pp.109.137935 19605552 10.1104/pp.109.137935 PMC2773056

[52] Ferro M, Brugière S, Salvi D, Seigneurin-Berny D, Court M, Moyet L, et al AT_CHLORO, a comprehensive chloroplast proteome database with subplastidial localization and curated information on envelope proteins. Mol Cell Prot. 2010;9:1063–1084. 10.1074/mcp.M900325-MCP200 PMC2877971 20061580

[53] Jaquinod M, Villiers F, Kieffer-Jaquinod S, Hugouvieu V, Bruley C, Garin J, et al A proteomics dissection of *Arabidopsis thaliana* vacuoles isolated from cell culture. Mol Cell Prot. 2007;6:394–412. 10.1074/mcp.M600250-MCP200 PMC2391258 17151019

[54] Shimaoka T, Ohnishi M, Sazuka T, Mitsuhashi N, Hara-Nishimura I, Shimazaki K-I, et al Isolation of intact vacuoles and proteomic analysis of tonoplast from suspension-cultured cells of *Arabidopsis thaliana*. Plant Cell Physiol. 2004;45:672–683. 15215502 10.1093/pcp/pch099

[55] Kusumoto KI, Matsushita-Morita M, Furukawa I, Suzuki S, Yamagata Y, Koide Y, et al Efficient production and partial characterization of aspartyl aminopeptidase from *Aspergillus oryzae*. J Appl Micro. 2008;105:1711–1719. 10.1111/j.1365-2672.2008.03889.x 18828788

[56] Lee S, Kim JS, Yun CH, Chae HZ, Kim K. Aspartyl aminopeptidase of *Schizosaccharomyces pombe* has a molecular chaperone function. BMB Reports. 2009;42:812–816. 20044953 10.5483/bmbrep.2009.42.12.812

[57] Carrie C, Kühn K, Murcha MW, Duncan O, Small ID, O’Toole N, et al Approaches to defining dual-targeted proteins in Arabidopsis. Plant J. 2009;57:1128–1139. doi: 10.1111/j.1365-313X.2008.03745.x 19036033 10.1111/j.1365-313X.2008.03745.x

[58] Small I, Wintz H, Akashi K, Mireau H. Two birds with one stone: Genes that encode products targeted to two or more compartments. Plant Mol Biol. 1998;38:265–277. 9738971

[59] Suzuki K, Nakamura S, Morimoto M, Fujii K, Noda NN, Inagaki F, et al Proteomic profiling of autophagosome cargo in *Saccharomyces cerevisiae*. PLoS ONE. 2014;9:e91651 doi: 10.1371/journal.pone.0091651 24626240 10.1371/journal.pone.0091651 PMC3953483

[60] Yuga M, Gomi K, Klionsky DJ, Shintani T. Aspartyl aminopeptidase is imported from the cytoplasm to the vacuole by selective autophagy in *Saccharomyces cerevisiae*. J Biol Chem. 2011;286:13704–13713. doi: 10.1074/jbc.M110.173906 21343297 10.1074/jbc.M110.173906 PMC3075714

[61] Michaeli S, Galili G, Genschik P, Fernie AR, Avin-Wittenberg T. Autophagy in plants—What's new on the menu? Trends Plant Sci. 2016;21:134–144. doi: 10.1016/j.tplants.2015.10.008 26598298 10.1016/j.tplants.2015.10.008

[62] Hirsch-Hoffmann M, Gruissem W, Baerenfaller K. pep2pro: the high-throughput proteomics data processing, analysis, and visualization tool. Front Plant Sci. 2012;3. 10.3389/fpls.2012.00123 PMC3371593 22701464

[63] Goda H, Sasaki E, Akiyama K, Maruyama-Nakashita A, Nakabayashi K, Li W, et al The AtGenExpress hormone and chemical treatment data set: experimental design, data evaluation, model data analysis and data access. Plant J. 2008;55:526–542. 18419781 10.1111/j.0960-7412.2008.03510.x

[64] Kilian J, Whitehead D, Horak J, Wanke D, Weinl S, Batistic O, et al The AtGenExpress global stress expression data set: protocols, evaluation and model data analysis of UV-B light, drought and cold stress responses. Plant J. 2007;50:347–363. doi: 10.1111/j.1365-313X.2007.03052.x 17376166 10.1111/j.1365-313X.2007.03052.x

[65] Schmid M, Davison TS, Henz SR, Pape UJ, Demar M, Vingron M, et al A gene expression map of *Arabidopsis thaliana* development. Nat Genet. 2005;37:501–506. doi: 10.1038/ng1543 15806101 10.1038/ng1543

[66] Toufighi K, Brady SM, Austin R, Ly E, Provart NJ. The Botany Array Resource: e-Northerns, expression angling, and promoter analyses. Plant J. 2005;43:153–163. doi: 10.1111/j.1365-313X.2005.02437.x 15960624 10.1111/j.1365-313X.2005.02437.x

[67] Watanabe J, Tanaka H, Akagawa T, Mogi Y, Yamazaki T. Characterization of *Aspergillus oryzae* aspartyl aminopeptidase expressed in *Escherichia coli*. Biosci Biotech Biochem. 2007;71:2557–2560. 10.1271/bbb.70107 17928682

[68] Barrett AJ. Classification of peptidases. Methods Enzymol. 1994;244:1–15. 7845199 10.1016/0076-6879(94)44003-4

[69] Shen J, Zeng Y, Zhuang X, Sun L, Yao X, Pimpl P, et al Organelle pH in the Arabidopsis endomembrane system. Mol Plant. 2013;6:1419–1437. doi: 10.1093/mp/sst079 23702593 10.1093/mp/sst079

[70] Lauterbach SB, Coetzer TL. The M18 aspartyl aminopeptidase of *Plasmodium falciparum* binds to human erythrocyte spectrin in vitro. Malaria J. 2008;7. 10.1186/1475-2875-7-161 PMC2543045 18721457

[71] Nakamura Y, Inloes JB, Katagiri T, Kobayashi T. Chondrocyte-specific microRNA-140 regulates endochondral bone development and targets DNPEP to modulate bone morphogenetic protein signaling. Mol Cell Biol. 2011;31:3019–3028. doi: 10.1128/MCB.05178-11 21576357 10.1128/MCB.05178-11 PMC3133397

[72] Lee A, Slattery C, Nikolic-Paterson DJ, Hryciw DH, Wilk S, Wilk E, et al Chloride channel ClC-5 binds to aspartyl aminopeptidase to regulate renal albumin endocytosis. Am J Physiol-Renal Physiol. 2015;308:F784–F792. doi: 10.1152/ajprenal.00322.2014 25587118 10.1152/ajprenal.00322.2014

[73] Farrokhi N, Whitelegge JP, Brusslan JA. Plant peptides and peptidomics. Plant Biotech J. 2008;6:105–134. 10.1111/j.1467-7652.2007.00315.x 18069950

[74] Wheeler JI, Irving HR. Evolutionary advantages of secreted peptide signalling molecules in plants. Func Plant Biol. 2010;37:382–394.

[75] Graciet E, Wellmer F. The plant N-end rule pathway: structure and functions. Trends Plant Sci. 2010;15:447–453. doi: 10.1016/j.tplants.2010.04.011 20627801 10.1016/j.tplants.2010.04.011

[76] Tyedmers J, Mogk A, Bukau B. Cellular strategies for controlling protein aggregation. Nat Rev Mol Cell Biol. 2010;11:777–788. doi: 10.1038/nrm2993 20944667 10.1038/nrm2993

[77] DuPrez K, Scranton M, Walling Linda L, Fan L. Structural basis for the chaperone activity enhancement by mutation K354E in tomato acidic leucine aminopeptidase. Acta Crystallogr D—Biol Crystallogr. 2016;72:694–702. 10.1107/S205979831600509X 27139632

[78] Gu YQ, Walling LL. Identification of residues critical for activity of the wound-induced leucine aminopeptidase (LAP-A) of tomato. Eur J Biochem. 2002;269:1630–1640. 11895433 10.1046/j.1432-1327.2002.02795.x

[79] DuPrez K, Scranton M, Walling Linda L, Fan L. Crystal structure of tomato wound-induced leucine aminopeptidase sheds light on its substrate properties. Acta Crystallogr D—Biol Crystallogr. 2014;70:1649–1658. 24914976 10.1107/S1399004714006245

