# Supplementary material for: Chlorophyte aspartyl aminopeptidases: Ancient origins, expanded families, new locations, and secondary functions
Source: PLoS One. 2017 Oct 12;12(10):e0185492. doi: 10.1371/journal.pone.0185492 (PMC5638241; doi:10.1371/journal.pone.0185492)
Supplement: S1 Table — (DOCX) [file pone.0185492.s001.docx]

**Table S1. Roles of conserved residues in the Arabidopsis, human, bovine*, P. aeruginosa*, and *Plasmodium* DAPs.**

| **AtDAP1** | **AtDAP2** | **PFM18AAP^1^** | **PaAP** | **HsDNPEP^2^** | **BtDNPEP^3^** | **Impact of mutation on activity** | **Role of residue** | **References** |
| --- | --- | --- | --- | --- | --- | --- | --- | --- |
| H22 | H82 | L23 (L24) | H21 | H43 (H33) | H44 (H19) | Decrease k_cat_  No change in K_M_ |  | ([1](#_ENREF_1),[2](#_ENREF_2)) |
| H83 | H143 | H86 (H87) | H82 | H104 (H94) | H115 (H90) | Inactive | Metal coordination (Zn2); part of the HDEDH motif | ([1](#_ENREF_1),[3-5](#_ENREF_3)) |
| S86 | S146 | S89 (S90) | S84 | S104 (S97) | S143 (S118) |  | Interacts with D260 and H2O to enable coordination of Zn1 and Zn2 | ([4](#_ENREF_4)) |
| H161 | H218 | H160 (H161) | H156 | H180 (H170) | H191 (H166) | Inactive | His is located within a loop that constrains the size of the active site and interacts with opposing subunit in dimer; proposed to be critical for peptide hydrolysis | ([1](#_ENREF_1),[3-5](#_ENREF_3)) |
| D264 | D310 | D324 (D325) | D236 | D274 (D264) | D285 (D260) | Decrease k_cat_  No change in K_M_ | Metal coordination (Zn1 & Zn2); part of the HDEDH motif; has cis conformational bond with N286 (bovine) important for orientation of Asp260 for metal coordination with Ser 118 | ([2-5](#_ENREF_2)) |
| N265 | N311 | N325 (N326) | N237 | N275 (N265) | N286 (N261) |  | Forms a *cis* conformational bond with neighboring Asp260 | ([4](#_ENREF_4)) |
| E301 | E347 | E379 (E380) | E265 | E311 (E301) | E322 (E297) |  | Catalytic pocket; binds solvent or h20 or ABH | ([2-5](#_ENREF_2)) |
| E302 | E348 | E380 (E381) | E266 | E312 (E302) | E323 (E298) |  | Metal coordination (Zn1); part of the HDEDH motif | ([2-5](#_ENREF_2)) |
| D347 | D396 | D344 (D435) | D307 | D356 (D346) | D367 (D342) |  | Metal coordination (Zn2); part of the HDEDH motif; binds substrate | ([3-5](#_ENREF_3)) |
| M348 | M397 | M435 (M436) | D308 | M357 (M347) | M368 (M343) |  | Substrate-binding pocket  H20 bonds with Asp carboxylate | ([5](#_ENREF_5)) |
| H350 | H399 | H437 (H438) | H310 | H359 (H349) | H370 (H345) | Decrease k_cat_  No change in K_M_ | Substrate-binding pocket; part of the HDEDH motif; binds substrate and thought to be important for substrate preference | ([1](#_ENREF_1),[3-5](#_ENREF_3)) |
| H353 | H402 | H440 (H441) | H313 | H362 (H352) | H373 (H348) | Multimer dissociates Lowers k_cat_; inactive | Multimer association | ([1](#_ENREF_1)) |
| H360 | H409 | V447 (V448) | H320 | H369 (H359) | H380 (H355) | Decrease k_cat_ |  | ([1](#_ENREF_1)) |
| H364 | H413 | H451 (H452) | H324 | H373 (H363) | H384 (H359) | Decrease k_cat_ | Lines catalytic channel | ([1](#_ENREF_1),[3](#_ENREF_3)) |
| K375 | K424 | K462 (K463) | K335 | K384 (K374) | K395 (K370) |  | At end of substrate-binding pocket and determines specificity for acidic residues; binds substrate (Glu & Asp) carboxylate residues; binds ABH | ([2-5](#_ENREF_2)) |
| Y382 | Y431 | Y469 (Y470) | Y342 | Y391 (Y381) | Y402 (Y377) |  | Substrate-binding pocket; binds substrate (Glu); Binds ABH | ([3](#_ENREF_3),[5](#_ENREF_5)) |
| F406 | Y455 | Y499 (Y500) | F366 | L410 (L400) | L426 (L401) |  | Substrate-binding pocket | ([5](#_ENREF_5)) |
| G415 | G464 | G508 (G509) | G375 | G424 (G414) | G435 (G410) |  | Catalytic pocket | ([5](#_ENREF_5)) |
| S416 | S465 | S509 (S510) | S376 | T425 (T415) | T436 (T411) |  | Catalytic pocket | ([5](#_ENREF_5)) |
| T417 | T466 | T510 (T511) | T377 | T426 (T416) | T437 (T412) |  | Substrate-binding pocket | ([5](#_ENREF_5)) |
| M440 | M489 | M533 (M534) | M400 | M449 (M439) | M460 (M435) |  | Binds bridging solvent between Zn1 and Zn2 | ([5](#_ENREF_5)) |
| H441 | H490 | H534 (H535) | H401 | H450 (H440) | H461 (H436) | Decrease k_cat_  No change in K_M_ | Metal coordination (Zn1); part of the HDEDH motif | ([1-5](#_ENREF_1)) |
| ^1^ The *P. falciparum* 3D7 sequence has an N-terminal Met and is one residue shorter than the *P. falciparium* protein (which lacks a N-terminal Met) used for X-ray structure determination; residues in parentheses are the coordinates from the crystal structure (4EME_A) ([5](#_ENREF_5)).  ^2^ The human DNPEP is the full length protein (Q9ULAO). The residue numbers for the mature protein are in parentheses and are 10 residues shorter ([1](#_ENREF_1),[3](#_ENREF_3)).  ^3^ The bovine DNPEP full-length protein is shown. The mature protein is 25 residues shorter (in parentheses). The mature protein was used for crystal structure determination ([4](#_ENREF_4)). | | | | | | | | |

1. Wilk, S., E. Wilk, and R.P. Magnusson. (2002). Identification of histidine residues important in the catalysis and structure of aspartyl aminopeptidase. Archives of Biochemistry and Biophysics 407: 176-183.

2. Nguyen, D.D., R. Pandian, D. Kim, S.C. Ha, H.-J. Yoon, K.S. Kim, K.H. Yun, J.-H. Kim, and K.K. Kim. (2014). Structural and kinetic bases for the metal preference of the M18 aminopeptidase from *Pseudomonas aeruginosa*. Biochemical and Biophysical Research Communications 447: 101-107.

3. Chaikuad, A., E.S. Pilka, A. De Riso, F. von Delft, K.L. Kavanagh, C. Venien-Bryan, U. Oppermann, and W.W. Yue. (2012). Structure of human aspartyl aminopeptidase complexed with substrate analogue: insight into catalytic mechanism, substrate specificity and M18 peptidase family. BMC Structural Biology 12: 14 doi. 10.1186/1472-6807-12-14.

4. Chen, Y.Y., E.R. Farquhar, M.R. Chance, K. Palczewski, and P.D. Kiser. (2012). Insights into substrate specificity and metal activation of mammalian tetrahedral aspartyl aminopeptidase. Journal of Biological Chemistry 287: 13356-13370.

5. Sivaraman, K.K., C.A. Oellig, K. Huynh, S.C. Atkinson, M. Poreba, M.A. Perugini, K.R. Trenholme, D.L. Gardiner, G. Salvesen, M. Drag, J.P. Dalton, J.C. Whisstock, and S. McGowan. (2012). X-ray crystal structure and specificity of the *Plasmodium falciparum* malaria aminopeptidase PfM18AAP Journal of Molecular Biology 422: 495-507.
