# Supplementary material for: Chlorophyte aspartyl aminopeptidases: Ancient origins, expanded families, new locations, and secondary functions
Source: PLoS One. 2017 Oct 12;12(10):e0185492. doi: 10.1371/journal.pone.0185492 (PMC5638241; doi:10.1371/journal.pone.0185492)
Supplement: S2 Table — (DOCX) [file pone.0185492.s002.docx]

| **Table S2. Musite-predicted Ser and Thr phosphorylation sites in plant DAP1 and DAP2 proteins** | | | | | |
| --- | --- | --- | --- | --- | --- |
| **Division** | **Protein** | **DAP protein regions/domains and Musite-predicted phosphorylated residues^A^** | | | |
|  |  | **Transit Peptide^B^** | **Proteolytic**  **Domain 1** | **Dimerization Domain** | **Proteolytic**  **Domain 2** |
| eudicot | AtDAP1 | --- |  | Thr194 | Ser 282, **Ser284,** Ser313 |
| eudicot | GmDAP1a | --- |  |  | Ser294 |
| eudicot | GmDAp1b | --- |  |  | Ser294 |
| eudicot | MtDAP1 | --- |  | Ser202 | Ser287, Ser 289, Ser 318 |
| eudicot | PtDAP1a | --- |  |  | Ser294, Ser296, Ser317, Ser325 |
| eudicot | PtDAP1b | --- |  |  |  |
| eudicot | VvDAP1 | --- |  |  | Ser321 |
| monocot | BdDAP1 | --- |  |  | Ser311 |
| monocot | OsjDAP1.1 | --- |  |  | Ser305, Ser 313 |
| monocot | SbDAP1 | --- |  |  | Ser302, Ser310 |
| gymnosperm | PsDAP1 | --- |  |  | Ser336, Ser 348, Ser355 |
| eudicot | AtDAP2 | Ser57, Ser60 |  | Ser244, Ser247 |  |
| eudicot | GmDAP2a |  |  |  |  |
| eudicot | GmDAP2b |  |  |  |  |
| eudicot | MtDAP2 | Ser28 |  | Thr269 | Ser349, Ser350, Ser352 |
| eudicot | PtDAP2 | Ser17 |  | Ser244 |  |
| eudicot | VvDAP2 | Ser34, Ser71 |  |  |  |
| monocot | BdDAP2 | Ser21 |  | Ser243 |  |
| monocot | HvDAP2 | Ser21 |  | Ser236 |  |
| monocot | OsjDAP2 | Ser51, Ser61, Ser28 |  |  |  |
| monocot | SbDAP2 | Ser52 |  | Ser248, Ser244 |  |
| ^A^ Based on X-ray structures of the human, bovine and *Plasmodium* DAPs, the two regions that form the DAP proteolytic domain and the dimerization domain were determined for plant DAPs (Fig. 1). Musite was used to predict Ser and Thr residues with the potential for phosphorylation ([1](#_ENREF_1)). One AtDAP1’s Ser284 has been shown to be phosphorylated in vivo ([2](#_ENREF_2),[3](#_ENREF_3)); it is in bold and shaded in gray.  ^B^ The length of transit peptides were determined by TargetP or ChloroP (Table S1). A Ser residue that resides within or in close proximity to predicted transit peptide cleavage sites are listed. DAP1 proteins do not have transit peptides; this is indicated by dashes (---). | | | | | |

1. Yao, Q., J. Gao, C. Bollinger, J. Thelen, and D. Xu. (2012). Predicting and analyzing protein phosphorylation sites in plants using Musite. Frontiers in Plant Science 3: Article 186.

2. Sugiyama, N., H. Nakagami, K. Mochida, A. Daudi, M. Tomita, K. Shirasu, and Y. Ishihama. (2008). Large-scale phosphorylation mapping reveals the extent of tyrosine phosphorylation in Arabidopsis. Molecular Systems Biology 4: 193 doi:10.1038/msb.2008.32.

3. Durek, P., R. Schmidt, J.L. Heazlewood, A. Jones, D. Maclean, A. Nagel, B. Kersten, and W.X. Schulze. (2010). PhosPhAt: the *Arabidopsis thaliana* phosphorylation site database. An update. Nucleic Acids Research 38: D828-D834.
