# Supplementary material for: Chlorophyte aspartyl aminopeptidases: Ancient origins, expanded families, new locations, and secondary functions
Source: PLoS One. 2017 Oct 12;12(10):e0185492. doi: 10.1371/journal.pone.0185492 (PMC5638241; doi:10.1371/journal.pone.0185492)
Supplement: S3 Table — (PDF) [file pone.0185492.s003.pdf]

Table S3. Chlorophyte *DAP* genes and *DAPs* from other selected species.

Class/Division	Species	Gene Name	Locus Name	Chromosome	Accession(s)	Predicted Localization [A]					# residues (total)
						Plant-mLoc	TargetP (reliability class)	Predotar	ChloroP	Transit peptide length (# residues)	
Embryophyta (eudicot)	*Arabidopsis lyrata*	*AlDAP1*	ARALYDRAFT_332278		XP_002866374.1	Cp, C or M	Other (RC3)	Other	--	--	477
Embryophyta (eudicot)	*Arabidopsis lyrata*	*AlDAP2*	ARALYDRAFT_487250		XP_002873165.1	Cp	Cp (RC3)	Cp	Cp	47 (47)	526
Embryophyta (eudicot)	*Arabidopsis thaliana*	*AtDAP1*	At5g60160	Chr 5	NM_200824.1	Cp	Other (RC4)	Other	--	--	477
Embryophyta (eudicot)	*Arabidopsis thaliana*	*AtDAP2*	At5g04710	Chr 5	NP_196091.1	Cp	Cp (RC1)	Cp	Cp	63 (63)	526
Embryophyta (eudicot)	*Glycine max*	*GmDAP1a*	LOC100776903	Chr 4	XP_003523136.1	Cp, C or M	Other (RC3)	Other	--	--	487
Embryophyta (eudicot)	*Glycine max*	*GmDAP1b*	LOC100781379	Chr 6	XP_003526970.1	Cp or C	Other (RC3)	Other	--	--	487
Embryophyta (eudicot)	*Glycine max*	*GmDAP2a*	LOC100790595	Chr 20	XP_003556268.1	Cp	Cp (RC1)	Cp	Cp	47 (45)	518
Embryophyta (eudicot)	*Glycine max*	*GmDAP2b*	LOC100788773	Chr 10	XP_003535505.1	Cp	Cp (RC1)	Cp	Cp	51 (47)	522
Embryophyta (eudicot)	*Medicago truncatula*	*MtDAP1*	MTR_3g088860	Chr 3	XP_003602076.1	Cp, C or M	Other (RC2)	Other	--	--	482
Embryophyta (eudicot)	*Medicago truncatula*	*MtDAP2*	MTR_1g092920	Chr 1	XP_003591776.1	Cp	Cp (RC1)	Cp	Cp	48 (48)	550
Embryophyta (eudicot)	*Populus trichocarpa*	*PtDAP1a*	POPTRDRAFT_835204	Chr LGXV	XP_002321687.1	Cp or C	Other (RC2)	Other	---	--	489
Embryophyta (eudicot)	*Populus trichocarpa*	*PtDAP1b* [B]	POPTRDRAFT_834353	Chr LGXII	XP_002318119.1, DT507864	Cp or C	Other (RC4)	Other	---	--	483
Embryophyta (eudicot)	*Populus trichocarpa*	*PtDAP2*	POPTRDRAFT_420959	Chr LGVIII	XP_002311016.1	Cp	Cp (RC2)	Cp	Cp	55 (51)	526
Embryophyta (eudicot)	*Vitis vinifera*	*VvDAP1*	LOC100249091	Chr 17	XP_002283475.1	Cp, C or M	Other (RC2)	Other	---	--	485
Embryophyta (eudicot)	*Vitis vinifera*	*VvDAP2*	LOC100265348	Chr 13	XP_002266672.1	M or Cp	Cp (RC5)	Other	Cp	64 (64)	535
Embryophyta (monocot)	*Brachypodium distachyon*	*BdDAP1*	Bradi4g39590; LOC100820851	Chr 4	P_003578741.1	Cp	Other (RC3)	Other	--	--	475
Embryophyta (monocot)	*Brachypodium distachyon*	*BdDAP2*	LOC100830808	Chr 2	XP_003565148.1	M	M (RC3)	M	Cp	110 (30)	520
Embryophyta (monocot)	*Hordeum vulgare*	*HvDAP1*	partial cDNA only		BAK02635.1	--	--	--	--		FL192719
Embryophyta (monocot)	*Hordeum vulgare*	*HvDAP2* [B]	AK355479.1 (cDNA only)		AK355479.1	Cp or M	M (RC5)	M	Cp	103 (41)	513
Embryophyta (monocot)	*Oryza sativa japonica*	*OsjDAP1.1* [C]	LOC_Os12g13390.1	Chr 12	NP_001066463.1	Cp	Other (RC4)	--	Other	--	478
Embryophyta (monocot)	*Oryza sativa japonica*	*OsjDAP1.2* [C]	LOC_Os12g13390.2	Chr 12	NP_001066463.1	Cp, C or M	Other (RC4)	--	Other	--	408
Embryophyta (monocot)	*Oryza sativa japonica*	*OsjDAP1.3* [C]	LOC_Os12g13390.3	Chr 12	NP_001066463.1	Cp	Other (RC4)	--	Other	--	320
Embryophyta (monocot)	*Oryza sativa japonica*	*OsjDAP2* [D]	LOC_Os01g73680.1	Chr 1	NP_001045513.1	M	M (RC4)	M	Cp	41 (57)	525
Embryophyta (monocot)	*Sorghum bicolor*	*SbDAP1*	Sb_03g029130	Chr 3	XP_002458215.1	Cp or C	Other (RC3)	Other	--	--	475
Embryophyta (monocot)	*Sorghum bicolor*	*SbDAP2*	Sb_03g047100	Chr 3	XP_002457006.1	M	M (RC5)	M	Cp	115 (45)	524
Embryophyta (monocot)	*Zea mays*	*ZmDAP1*	LOC100304311		ACN26704.1	Cp	Other (RC3)	Other	--	--	475
Embryophyta (monocot)	*Zea mays*	*ZmDAP2* [B]	assembled from ESTs		FL192718; FL192719	N	M (RC4)	M	Cp	115 (54)	155
Embryophyta (gymnosperm)	*Picea sitchensis*	*PsDAP1*	ABR17053		EF677212.1	Cp or C	Other (RC3)	Other	--	--	502
Lycopodiophyta (clubmoss)	*Selaginella moellendorffii*	*SmDAP1a*	SELMODRAFT_80579		XP_002963681.1	Cp	Sp (RC5)	Other	--	--	475
Lycopodiophyta (clubmoss)	*Selaginella moellendorffii*	*SmDAP1b*	SELMODRAFT_101663		XP_002974770.1	Cp or C	Other (RC4)	Other	--	--	519
Lycopodiophyta (clubmoss)	*Selaginella moellendorffii*	*SmDAP2a* [E]	SELMODRAFT_442726		XP_002975004.1	Cp or M	Other (RC2)	Other	--	--	477
Lycopodiophyta (clubmoss)	*Selaginella moellendorffii*	*SmDAP2b* [F]	SELMODRAFT_86972		XP_002967289.1	Cp	Other (RC4)	Other	--	--	519
Bryophyta (moss)	*Physcomitrella patens subsp. patens*	*PpDAP1*	PHYPADRAFT_118420		XP_001756754.1	Cp	Other (RC3)	Other	--	--	467
Bryophyta (moss)	*Physcomitrella patens subsp. patens*	*PpDAP2a*	PHYPADRAFT_111408		XP_001751765.1	Cp	Cp (RC4)	Cp	--	--	586
Bryophyta (moss)	*Physcomitrella patens subsp. patens*	*PpDAP2b1*	PHYPADRAFT_191408		XP_001773362.1	Cp	Other (RC2)	Other	--	--	486
Bryophyta (moss)	*Physcomitrella patens subsp. patens*	*PpDAP2b2*	PHYPADRAFT_154257		XP_001784619.1	Cp	M (RC5)	Other	--	22 (--)	496
Chlorophyta (green algae)	*Chlamydomonas reinhardtii* [k]	*CrDAP*	AAP1	Chr 10	XP_001690598.1						565
Chlorophyta (green algae)	*Micromonas pusilla CCMP1545*	*MpCCMP DAP2*	MICPUCDRAFT_43781		XP_003055398.1						476
Chlorophyta (green algae)	*Micromonas pusilla CCMP1545*	*MpCCMPDAP*	MICPUCDRAFT_1995		XP_003060266.1						444
Chlorophyta (green algae)	*Micromonas pusilla sp. RCC299*	*MpRccDAP2*	MICPUN_62784	Chr 11	XP_002505350.1						500
Chlorophyta (green algae)	*Micromonas pusilla sp. RCC299*	*MpRccDAP*	MICPUN_55052	Chr 1	XP_002507546.1						495
Chlorophyta (green algae)	*Ostreococcus lucimarinus CCE9901*	*OlDAP2* [G]	OSTLU_13136	Chr 12	XP_001420830.1						465
Chlorophyta (green algae)	*Ostreococcus lucimarinus CCE9901*	*OlDAP*	OSTLU_27563	Chr 14	XP_001421486.1						512
Chlorophyta (green algae)	*Ostreococcus tauri*	*OtDAP2*	Ot12g02310	Chr 12	XP_003082377.1						486
Chlorophyta (green algae)	*Ostreococcus tauri*	*OtDAP* [H]	Ot15g01860	Chr 15	XP_003083329.1						1045
Chlorophyta (green algae)	*Chlorella variabilis*	*CvDAP*	CHLNDRAFT_143544		EFN57167.1						566
Chlorophyta (green algae)	*Coccomyxa subellipsoidea* C-169	*CsDAP*	COCSUDRAFT_15496		EIE23587.1						482
Chlorophyta (green algae)	*Volvox carteri f. nagariensis*	*VcDAPa* [I]	VOLCADRAFT_104613		XP_002950223.1						430
Chlorophyta (green algae)	*Volvox carteri f. nagariensis*	*VcDAPb* [I]	VOLCADRAFT_104511		XP_002949870.1						320
Glaucophyta (glaucophyte algae)	*Cyanophora paradoxa*	*CpDAP* [J]	Contig8853-abnit gene								396
Rhodophyta (red algae)	*Porphyridium cruentum*	*PcDAP* [K]	assembled								541

Rhodophyta (red algae)	*Cyanidioschyzon merolae*	*CmDAP*[L]	CMG039C	Chr7							524
Mammalia	*Mus musculus*	*MmDNPEP*	DNPEP	Chr 1	AAD01212.1						473
Mammalia	*Homo sapiens*	*HsDNPEP*	DNPEP	Chr 2	Q9ULA0.1; 4DYO						485
Mammalia	*Bos taurus*	*BtDNPEP*	DNPEP		3VAT						496
Oomycota	*Phytophthora infestans* T30-4	*PiDAP*	PITG_02114		XP_002907085						462
Ascomycota	*Saccharomyces cerevisiae*	*ScDAP (ScApe4)*	YHR113W	Chr VIII	NP_011981.1						490
Ascomycota	*Schizosaccharomyces pombe*	*SpDAP (SpAAP1)*	SPAC4F10.02	Chr I	NP_594745.1						467
Ascomycota	*Saccharomyces cerevisiae*	*ScAP1 (ScApeI; Lap4)*	CAA81943	Chr XI	P14904						514
Ascomycota	Aspergillus oryzae RIB40	*AorAP1 (Lap4)*	AOR_1_1000134		XP_001822749						510
Ascomycota	Aspergillus oryzae RIB40	*AorDAP (DNPEP)*	AOR_1_2506174		XP_001818370						498
Apicomplexa	*Plasmodium falciparum*	*PfM18AAP*	XM_001352153	Chr 9	XP_001352189						570
Archaea	*Aciduliprofundum boonei T469*	*AbM18peptidase*	Aboo_1300		ZP_04873979.1						471

[A] Protein localization predictions were made for all embryophyte, bryophyte, and lyopodiophyte DAPs using TargetP or Predotar. Transit peptide searches were not performed for the algae, oomycetes, Ascomycota, Archaea, or Mammalia DAPs. Chloroplast (Cp), mitochondria (M), endomembrane (Signal peptide, Sp), nucleus (N), C (cytosol), or another (Other) subcellular localization is indicated. Reliability class (RC) is indicated for the TargetP predictions. In some cases, Predotar predicted a possible localization to the mitochondrion [M(?)] or chloroplast [(C(?)]. When chloroplast localization was predicted, the transit peptide lengths predicted by Target P and ChloroP (in parentheses) are provided.

[B] A completeDAP sequence was not predicted by genome or full-length cDNA sequences. Sequence and EST databases were interrogated and an assemblage based on identities to other DAPs was made.

[C] Os12g13390 *(OsjDAP* 1) was previously known as Os12g0236500. POGS predicts three alternative transcripts from this gene. In addition, the 3' end of the Os01g73690 RNA, which is transcribed from the opposite DNA strand may encoded a natural antisense transcript for *OsDAP2.*

[D] Os1g73680 (*OsjDAP2*) was previously known as Os1g0967900.

[E] There are two *S. moellendorffii* cDNAs that predict a DAP2a sequence. SELMODRAFT_176342 encodes a protein identical to SELMODRAFT_442726 but appears to be truncated at the N-terminus. It is not clear if this is an alternative transcription initiation, an alternative splicing product or a truncated cDNA.

[F] There are two *S. moellendorffii* cDNAs that predict a DAP2b sequence. The SELMODRAFT_68972 and SELMODRAFT_164111 are identical except for one residue substitution and a 3 residue insertion/deletion. It is unclear if this this a different gene or polymorphic cDNA.

[G] The *Ostreococcus lucimarinus DAP2* gene appears to encode a trucated DAP protein or is misannoted.

[H] The *Ostreococcus taurii* DAP protein is currently predicted to have a long C-terminal extension. It may be a novel fusion protein with an N-terminal M18 DAP domain and C-terminal ATPase domain or OtDAP-like may be misannotated.

[I] The analyses of *V. carteri* DAP cDNA ORFs indicated that the two DAP cDNAs have one or more multiple indels that cause frameshifts. Neither clone encodes a full-length DAP protein.

[J] The *C .paradoxa* DAP was identified by BLAST analyses of the *Cyanophora paradoxa* genome initiative database at http://cyanophora.rutgers.edu/cyanophora/home.php.

[K] The *P. cruentum* DAP sequence was assembled from four contigs (Contig 1476, GCDJ7DB01EK2W7, Contig 12535, GCDJ7DB01DVTHH) provided by Dr. Hwan Su Yoon (Sungkyunkwan University, Korea). This gene product should be considered tentative. Four single nt substituions and one region with a cluster of four nucleotide differences were detected during the assembly. In all cases the nucleotides that optimized alignments with the Micrococcus DAP and A. thaliana DAP1 were chosen. It is not known if translation begins at the first or second Met in the assembled sequence.

[L] The *C.merole* DAP was identified by BLAST analyses of the database at http://merolae.biol.s.u-tokyo.ac.jp
